# PDMS Membranes Drilled by Proton Microbeam Writing: A Customizable Platform for the Investigation of Endothelial Cell–Substrate Interactions in Transwell-like Devices

**DOI:** 10.3390/jfb16080274

**Published:** 2025-07-28

**Authors:** Vita Guarino, Giovanna Vasco, Valentina Arima, Rosella Cataldo, Alessandra Zizzari, Elisabetta Perrone, Giuseppe Gigli, Maura Cesaria

**Affiliations:** 1 CNR NANOTEC—Institute of Nanotechnology, c/o Campus Ecotekne, 73100 Lecce, Italy; vita.guarino@unisalento.it (V.G.); alessandra.zizzari@cnr.it (A.Z.); elisabetta.perrone@cnr.it (E.P.); giuseppe.gigli@unisalento.it (G.G.); 2 Department of Experimental Medicine, University of Salento, 73100 Lecce, Italy; 3 Department of Mathematics and Physics “Ennio De Giorgi”, University of Salento, Campus Ecotekne, Via per Arnesano, 73100 Lecce, Italy; giovanna.vasco@unisalento.it (G.V.); rosella.cataldo@unisalento.it (R.C.)

**Keywords:** proton beam writing, transwell-like device, endothelial cells, PDMS membranes

## Abstract

Cell migration assays provide valuable insights into pathological conditions, such as tumor metastasis and immune cell infiltration, and the regenerative capacity of tissues. In vitro tools commonly used for cell migration studies exploit commercial transwell systems, whose functionalities can be improved through engineering of the pore pattern. In this context, we propose the fabrication of a transwell-like device pursued by combining the proton beam writing (PBW) technique with wet etching onto thin layers of polydimethylsiloxane (PDMS). The resulting transwell-like device incorporates a PDMS membrane with finely controllable pore patterning that was used to study the arrangement and migration behavior of HCMEC/D3 cells, a well-established human brain microvascular endothelial cell model widely used to study vascular maturation in the brain. A comparison between commercial polycarbonate membranes and the PBW-holed membranes highlights the impact of the ordering of the pattern and porosity on cellular growth, self-organization, and transmigration by combining fluorescent microscopy and advanced digital processing. Endothelial cells were found to exhibit distinctive clustering, alignment, and migratory behavior close to the pores of the designed PBW-holed membrane. This is indicative of activation patterns associated with cytoskeletal remodeling, a critical element in the angiogenic process. This study stands up as a novel approach toward the development of more biomimetic barrier models (such as organ-on-chips).

## 1. Introduction

Cell migration is a pivotal biological process that orchestrates critical functions such as morphogenesis, tissue repair, and immune response regulation [1,2,3]. The deregulation of cell migration is implicated in a wide array of pathological conditions, including autoimmune disorders, chronic inflammatory diseases, and cancer, where it plays a key role in metastasis [4,5]. As a cornerstone of cell biology, embryology, immunology, and neuroscience, the study of cell migration has greatly advanced with breakthroughs in molecular biology, biochemistry, and cutting-edge imaging technologies [6,7,8,9].

A critical aspect of studying cell migration involves the development and use of in vitro models that allow for controlled and reproducible investigation of cellular behaviors [10,11,12]. These models are invaluable for uncovering the fundamental principles of cell migration and evaluating therapeutic strategies. One common method is the scratch wound assay, where a “wound” is created in a cell monolayer to observe how cells migrate to close the gap [13,14]. This approach is simple and effective for studying two-dimensional (2D) migration, although it does not fully replicate the three-dimensional (3D) tissue environment. Another widely used approach is the transwell assay technique, which involves the usage of porous membranes, with or without an extracellular matrix, to study cell movement in response to chemical gradients [12,15]. While effective for studying chemotaxis and invasion, this model is limited by its static and two-dimensional nature.

Organ-on-chip (OoC) systems represent an advanced frontier in the field of microfluidic models in that they dynamically reproduce tissue-like structures, hence allowing cell migration to be studied under conditions that closely resemble the in vivo environment [16,17,18]. In this framework, porous membranes are valuable OoC components for in vitro cell culture experiments aimed at gaining functional insight into biological signaling mechanisms, cell–cell communication, and predicting clinical responses to drug screening. In tissue barrier and cellular co-culture models, porous membranes enable physical and biochemical crosstalk between cells [19].

Microporous membranes usually include relatively large continuous (non-porous) regions in between pores, over which cells adhere and proliferate, which are detrimental for nutrient and oxygen uptake [20]. Indeed, the microenvironment characteristics (size, density, and random versus ordered distribution of the pores, supply of oxygen, and nutrients) affect the attachment, proliferation, differentiation, and viability of the cells [21]. Randomly distributed pores and non-uniform in-depth structure of the pores introduce nonoptimal conditions for uniform cell proliferation and transmigration. Uncontrolled cell dynamics make it difficult to regulate the interplay between in-plane metabolism and the migration across the pores of the cells. To recover proper oxygen uptake, possible solutions are increasing the permeability of the substrate by changing the preparation of the polymeric material or inserting nanopores to increase the porosity of the membrane [22]. Since all this can impact negatively on the structural integrity of the membrane and cause its damaging while integrating it into cell culture devices, a proper choice of the materials and a robust fabrication approach able to tune pore size and interpore spacing in a reproducible way is highly desirable.

Most OoC devices for disease modeling and therapeutic studies are manufactured in polydimethylsiloxane (PDMS) because of its low elastic modulus making it similar to the biological membranes of soft tissues, low stiffness, tunable oxygen permeability, chemical inertness, and well-known biocompatibility [23]. In order to separate two microfluidic compartments and enable cellular migration, most OoC devices employ commercially available track-etched polycarbonate (PC) or polyethylene terephthalate (PET) 0.4–8 µm pore-sized membranes. The advantage of these polymers is that they can be easily integrated into PDMS microfluidic devices through an appropriate functionalization protocol [24]. On the other hand, the track-etching fabrication method of the common porous membranes results in low pore density with regional variations [19]. Some research groups have chosen to fabricate PDMS porous membranes to create monolithic homogeneous chips to enhance the mechanical stability and reduce leakage disadvantages [25,26]. Additionally, compared to polycarbonate (PC) and polyethylene terephthalate (PET) membranes, PDMS membranes offer high transparency, which advantageously allows the real-time monitoring of cells during culture without labelling and is a prerequisite for high resolution imaging devoted to elucidating cellular events. Organ-on-chip models combined with advanced imaging technologies and automated analysis may constitute a novel approach for studying cell proliferation and migration in the perspective of health and disease implications [27,28,29].

Conventional microfabrication techniques, based on photolithography and dry etching, are commonly used to produce porous PDMS membranes due to their accuracy and control on the distribution of the pores [25,26,30]. Despite the relatively diffuse availability of these facilities, there are limitations related to the number of manufacturing steps and design flexibility.

In this study, we propose an alternative approach to produce porous PDMS membranes, which exploits the precise microfabrication characteristics offered by the proton microbeam writing (PBW) technique. Focused MeV-energy protons present several key advantages; they follow a straight-line path as they penetrate matter, exhibit minimal energy transfer, and cause negligible scattering during ion–electron collisions [31]. Previously, PBW has been successfully employed to fabricate smooth-sided vertical electrodes, molds, and stamps for replicating PDMS-based microfluidic chips [32,33]. Under our experimental conditions, PDMS was utilized as a positive resist; following exposure to a 3 MeV proton microbeam, PDMS became soluble in a developer and was subsequently removed using an appropriate wet etchant [34,35]. Leveraging this methodology, we report the design, implementation, and application of a PDMS-based transwell-like device including a PDMS membrane with a regular array of circularly shaped pores with diameter of 25 µm. Following the fabrication step, the transparent holed PDMS membrane was seeded with endothelial cells and imaged by optical microscopy. Light microscopy images were digitally processed by advanced methods to gain statistical information on the cell density and to investigate the influence of the patterned pore distribution on cell growth, cell–cell and cell–substrate interactions, cell–pore arrangement, and actin filament organization. This analysis step enables to go beyond the straightforward visual inspection of the microscopy images [36].

## 2. Materials and Methods

### 2.1. Materials

Soda-lime microscopic glass slides were provided by Pearl; Clevios PH 500 were purchased from Heraeus Clevios GmbH (Leverkusen, Germany) and toluene from J. T. Baker (Phillipsburg, NJ, USA). Sylgard-184, a two-part poly(dimethylsiloxane) (PDMS) elastomer, was purchased from Dow Corning (Midland, MI, USA). Sulfuric acid (H_2_SO_4_, 98%), sodium hydroxide (NaOH), hydrogen peroxide (H_2_O_2_, 30%), fibronectin bovine plasma, Phosphate Buffered Saline (PBS), formaldehyde, (3-Aminopropyl)triethoxysilane (APTES), phalloidin-TRITC and 4′,6-diamidino-2-phenylindole (DAPI), and 37% formaldehyde solution were purchased from Sigma-Aldrich/Merk (Milan, Italy). Triton X-100 was purchased from Invitrogen. Milli-Q water with a resistivity of 18.2 MΩ cm was used. PES syringe filters (0.45 μm) were purchased from Sartorius Stedim (Göttingen, Germany). Whatman^®^ Nuclepore™ Track-Etched PC membranes (pore size 8 μm) and Cover Glass (20 × 20 mm) were purchased from VWR International Srl (Milan, Italy).

### 2.2. Preparation of the PDMS-Holed Membranes by PBW

#### 2.2.1. Preparation of PDMS Films and PBW Experiments

To obtain a PDMS-holed membrane, the PBW technique was applied by using the nuclear microprobe beamline developed at CEDAD (Centre of Applied Physics, Dating and Diagnostics—University of Salento) [37]. Preliminary calibration experiments were performed to set the relevant parameters of a PBW experiment, which are fluence, current, and focusing conditions, by drawing different geometry patterns over PDMS films covering a glass substrate. The calibration experiments are not discussed herein, because they are out of the scope of the paper, and exhaustive literature reports are already available for the reader’s guidance [35]. We report on them in Section S1 of the Supplementary Materials.

To prepare PDMS films overlaying a glass substrate, a mixture of pre-polymer/curing agent (10:1 weight ratio) was diluted with toluene (4:1 in weight). Then, the solution was deposited by spin-coating and cured in an oven at 60 °C overnight to obtain a 10 µm-thick layer.

The drilled PDMS membranes were obtained by using a 3 MeV energy proton microbeam collimated and focused on purpose over a spot size of 25 μm diameter in vacuum (10^−7^ mbar) by using a triplet of quadrupole lenses (OM150, Oxford Microbeams Ltd., Granville Way, UK). The series of holes was carried out by stopping the beam with a Faraday Cup, while remotely moving the sample holder using the XYZ stepper motor package (Oxford Microbeams Ltd.).

The designed pore size of 25 µm was set inspired by the well-established literature knowledge and the investigated biological species, which are endothelial cells with an average diameter of 10–40 µm. Literature studies on the growth of endothelial cells on porous polymeric membranes as a function of the pore size and interpore spacing report the optimal pore size able to enhance the cell growth in vivo and in vitro [38]. In particular, pore size comparable to the cell size are indicated to favor cell migration on the opposite side of the membrane. Not only pore size but also interpore distance can affect endothelial cell growth. For investigating this aspect, we prepared PBW-holed membranes with varying interpore spacings from 50 to 190 µm and imaged the cell distributions (see Section 3.2 for results and discussion). To investigate differences with commercially available membranes, we selected a PC membrane with pores of 8 µm diameter. In practice, polymeric track-etched membranes are commercially available, as the culture inserts in multiwell plates with pore sizes ranging from 400 nm to 8 µm [19].

Noteworthy, PBW-holed PDMS membranes with 8 µm pores are not included in the discussion in this study, because the effective utility and impact on cell arrangement and transmigration of being able to design properly pore size and distribution can be clearly demonstrated under meaningful applicative conditions. Since the proton beam technique allows a control on pore size that is not possible otherwise, it is more meaningful accomplishing the optimal conditions in terms of pore size for the system under consideration rather than comparing identical geometrical conditions with the 8 µm constraint dictated by the limits of the common micro-fabrication techniques.

#### 2.2.2. Etching Protocol for Removing the Proton Beam Irradiated PDMS

Turning to the etching protocol, as far as we know, in the literature, the only chemical etching protocol of PDMS acting as a positive resist, following exposure to a 2 MeV proton microbeam, was developed quite recently [35]. Although we were inspired by such a literature source, wet etching of our PBW-treated PDMS samples was carried out based on a slightly different procedure. Indeed, different irradiation conditions and characteristics of the experimental set-up (see Section S1 of the Supplementary Materials for more detailed technical information) required a calibration of the chemical treatment protocol for the selective removal of the proton beam irradiated PDMS. The PDMS films overlaying the glass substrates were immersed into a NaOH (30%) solution at (80 ± 5) °C. After some preliminary tests, the etching time for obtaining the complete removal of the 10 µm thin PDMS layers in the areas treated by a 3 MeV proton microbeam was estimated to be 90 min.

### 2.3. Fabrication and Assembly of a Transwell-like Device Embedding the Membrane Patterned by PBW

**Figure 1:**
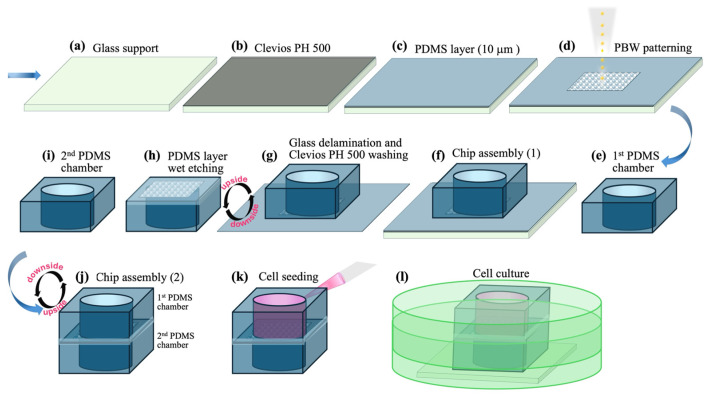
Fabrication scheme and assembly flow of the transwell chip embedding the PDMS holed membrane patterned by PBW. (**a**) Given a glass support, (**b**) a sacrificial layer of Clevios PH 550, and (**c**) a PDMS thin layer were spin-coated on it. (**d**) PBW-patterning on the PDMS membrane. (**e**) Fabrication of the first PDMS chamber and (**f**) its assembly with membrane/glass substrate after oxygen plasma treatment. (**g**) Immersion in water of the chip thus assembled and glass substrate delamination thanks to the sacrificial layer dissolution. (**h**) Membrane cutting along the edges to resizing it respect to the chamber dimensions and then its treatment with a NaOH-based etchant solution. (**i**–**l**) As a final step, assembly of the chamber integrating the membrane with a second PDMS chamber to allow (**k**) the cell seeding and then (**l**) the cell culture in a closed Petri dish placed into an incubator. See the text for more detailed explanation.

The production of a transwell-like device consisting of a PDMS membrane interposed in between two chambers was performed by combining several processing steps and techniques, as follows: PDMS polymerization, PBW of the PDMS thin layers according to a designed geometry, wet etching-based localized removal of PDMS modified by proton microbeam irradiation, and oxygen plasma activation for PDMS-to-PDMS bonding purposes. Figure 1 summarizes the main steps of the implemented procedure for assembling the transwell-like device under examination in this study.

First, the preparation of the PDMS template for the membranes was carried out according to the procedure reported elsewhere [39,40] and already used for cell culture applications [29,41].

Cleaning of a glass slide was accomplished by using piranha solution (3:1 H_2_SO_4_:H_2_O_2_), then washing with milli-Q water and drying under nitrogen flow. After cleaning the glass substrate, a thin layer of Clevios PH 500 solution (filtered through the PES syringe filter) acting as a sacrificial layer was deposited by spin coating followed by baking on a hot plate at 120 °C for 5 min. Thanks to its high solubility in water, this layer was used as a sacrificial layer to allow easy detachment of the PDMS membrane from the glassy support in the step of transwell device assembly.

A mixture of PDMS pre-polymer and curing agent (10:1 weight ratio) was diluted with toluene (4:1 in weight). Then, this solution was delivered on a glass substrate covered by the Clevios PH 500 layer and spin-coated to achieve a 10 µm-thick PDMS layer that was cured in an oven at 60 °C overnight.

Then, as above detailed, PBW patterning of the PDMS spin-coated layer was accomplished to draw pores of about 25 µm in diameter and variable periodicity.

Additionally, a 1.5 mm-thick PDMS layer was polymerized and cured in a flat Petri dish. From this layer, two slides of 1.5 cm × 1.5 cm were cut and punched in the middle to obtain a chamber of 8 mm in diameter. Then, the PDMS PBW-patterned membrane attached to the glass substrate was treated by oxygen plasma (Diener electronic) to produce hydroxyl groups that promote the adhesion between the membrane and the first PDMS chamber when they are put in conformal contact (chip assembly (1)). The chamber/membrane/glass substrate assembly was immersed in pure water to dissolve the sacrificial layer and remove the glassy support. Once substrate delamination occurred, the membrane/chamber assembly was treated with a NaOH (30%) solution at 80 °C for 90 min to dissolve/remove the regions modified due to proton microbeam irradiation and thus to realize the desired pores on the PDMS membrane. Finally, the etched membrane/chamber assembly was washed with pure water, dried with nitrogen flow, and treated by a further oxygen plasma step (on the other side of the membrane that was in contact with the sacrificial layer) and attached to the other PDMS chamber (chip assembly (2)). This procedure activates the membrane surface to the bonding to the second PDMS chamber to obtain the final device.

The first chamber, passing through the layer of PDMS, allows the seeding of the cells from the upper open side and the adhesion through the lower open side on the underlying membrane. The achieved transwell-like device was put onto a glass support and closed in a Petri dish for cell seeding and growth experiments, as better described below.

To investigate differences among different kinds of devices, two transwell-like devices with a PC membrane or a glass coverslip interposed in between two PDMS chambers were fabricated too. The PC membrane treated for 20 min at 80 °C with 5% APTES aqueous solution was sandwiched and bonded between the PDMS layers using oxygen plasma. The glass coverslip was simply interposed between the PDMS layers.

### 2.4. Cellular Growth in the Transwell Device and Cell Imaging

Human Cerebral Microvascular Endothelial Cells (HCMEC/D3, Cederlane, Burlington, NC, USA) were cultured in EndoGRO MV culture media (Millipore, Burlington, MA, USA) and used between passages 27 and 30. The devices (with PC membrane, glass coverslips and PDMS membrane) were sterilized by UV exposure for 30 min before the cell seeding. To allow the adhesion of HCMEC/D3 to the PDMS membrane, PC membrane, or glass coverslip, the microwell device was filled with a solution of 50 μg/mL fibronectin bovine plasma in PBS. The device was placed for 1 h at 37 °C into the cell incubator (5% CO_2_) to allow the fibronectin self-assembly on the membrane. HCMEC/D3 were then seeded into the microwell with the density of 3 × 10^5^ cells/cm^2^ and statically incubated for 24 h into the cell incubator (steps (m) (n) of Figure 1).

After 24 h of cellular culture in the fabricated device, cells were fixed with 3.7% formaldehyde in PBS (Phosphate Buffered Saline) for 10 min and, subsequently, permeabilized with 0.2% Triton X-100 in PBS for an additional time of 10 min. F-actin and nuclei staining was conducted using phalloidin-TRITC (tetramethylrhodamine isothiocyanate) and DAPI (4′,6-diamidino-2-phenylindole) with a concentration of 0.05 μg/mL and 0.1 μg/mL, respectively.

A fluorescence microscope (Nikon Europe B.V., Amstelveen, The Netherlands) was used to image both fixed and stained cells.

### 2.5. Image Processing and Statistical Analysis

Image processing was implemented on the light microscopy images based on different analysis tools as detailed hereafter.

(i) The light microscopy images associated with the phalloidin-TRITC and DAPI channels were processed by the two-dimensional structure tensor approach as implemented by the automated plugin OrientationJ (EPFL) [42] and the plugin StarDist [43]. available in the framework of the free-license software ImageJ-FiJi (version Version 1.54p 17 February 2025) [44].

OrientationJ implements the calculation of the structure tensor matrix associated with the gradient operator of the grey-level intensity function for each pixel of the image and along two principal independent spatial directions. The Riesz filter operator was used in our calculation due to its being translation-, rotation-, and scale-invariant as well as suitable to reduce the image noise and control the amplification of high frequencies [45,46]. Local orientational and anisotropy characteristics of the images were investigated by the spatial maps of energy E (i.e., the trace of the gradient structure tensor) and coherency [45,46].

In practice, while the gradient expresses the rate of change in an image with respect to any direction, the energy captures which features of the image are the most responsible for changes in the gradient and coherency characterizes the degree of elongation/orientation of the local features. Spatial energy maps exhibit a distribution of white-color regions (high energy pixels) and black-color regions (low energy pixels). In the case of coherency, C = 1 indicates high elongation (for instance, highly oriented regions and dominant local orientation), and C = 0 is associated with local isotropy.

(ii) The monogenic signal analysis was applied as implemented in the plugin MonogenicJ of ImageJ [47,48]. Given a 2D real signal I, the associated monogenic signal IM is a 3-valued vector with components given by the input signal I (for instance, an image), the real part or the Riesz transform of the input signal (Re (RI)), and the imaginary part of the Riesz transform of the input signal (Im (RI)). The quantities of interest associated with the monogenic signal are amplitude, orientation, and phase [49].

The peculiar advantages of the monogenic approach are the access to the local phase, phase-invariance of the monogenic amplitude, isotropy (rotation covariance), and representation of a D-dimensional signal (D ≥ 2) by introducing the directional Riesz transform.

Since the local phase provides a line/edge classification of contours (local contour type), it is associated with the local geometrical structure of the image. Noteworthy, the importance of local phase in image analysis (segmentation and edge-detection) is well established to be relevant because common gradient-based schemes for feature detection are unable to work properly in the presence of noise and/or changes in contrast [50].

(iii) The ImageJ/Fiji StarDist plugin was applied for detection and segmentation of nuclei imaged by fluorescence microscopy without preliminary thresholding of the source image [51].

(iv) In order to rigorously address any difference and impact on the cellular growth related to a commercial PC membrane versus our PBW-holed membrane, advanced texture and image classification approaches were applied, that is Principal Component Analysis (PCA) and Gray Level Co-Occurrence Matrix (GLCM). These methods work on the spatial arrangement of the gray-level values in the histogram associated with an image of interest.

The PCA approach relies on a linear change in basis on the data coordinates that maps a collection of N data in a real coordinate space to a set of sub-spaces with lower dimension without loss of information [52]. Indeed, the PCA analysis computes the more informative few principal components that retain as much of the data’s variation as possible and enables great simplification and easy visualization of the trends exhibited by large datasets.

Texture analysis based on the Gray Level Co-Occurrence Matrix (GLCM) approach was pioneered by Haralick et al. to provide high-level information on an image (color, shape, texture) in several biological and medical research contexts [53].

Twenty-three descriptors were calculated from each co-occurrence matrix evaluated at several angles (θ = {0°, 45°, 90°}) and with different distance d = {0, 2}, in order to make the results as independent as possible from the size of the sample [54]. We set other distances (d) too, but the considerations remain the same [55].

Since the PCA method is quite sensitive to the variances of the initial variables, a standardization (mean = 0 and variance = 1) of all the GLCM features was performed.

## 3. Results and Discussion

### 3.1. Wet Etching Protocol to Remove the Proton Beam Irradiated PDMS

Some preliminary tests aiming at the optimization of both PBW working conditions and etching parameters allowing the removal of the PDMS zones modified under proton microbeam irradiation were initially performed (details reported in Section S1). Selective etching occurs, because in the areas irradiated with highly energetic protons (typically 1–3 MeV), PDMS is damaged by generating radicals (from removal of the methyl group out of the –Si–O–Si– network, breaking of the –Si–O–Si– backbone chain with loss of volatile products). Thus, a process of radical rearrangements induces a decrease in the –Si–O–Si– short chain and an increase in the Si-O-Si long chain [34,35,56,57]. As a practical outcome, the significant degradation of the proton microbeam-treated area turns PDMS into a rigid glass-like material that causes its shrinking/compaction. As such, modified PDMS becomes more sensitive to alkali hydroxides (such as KOH or NaOH) than native PDMS, and it can be selectively removed in the irradiated regions by wet etching [35].

Lines were chosen for performing preliminary experiments, because they are easier than holes to be inspected visually by optical microscopy and by a profilometer to monitor the depth evolution during the etching treatment (Figure S2). In particular, a set of channels with a nominal width of ~25 μm and periodicity of 150 µm were written by a 3 MeV proton microbeam at low proton microbeam fluences (current of 6 pA and fluence of 6000 pC/mm^2^). These specimens were exploited for optimizing the etching experiments by changing the chemical composition of the etchant solution as well as the temperature and exposure time conditions. The etching protocol set-up for channels was then demonstrated to work properly to obtain PBW holes in PDMS membranes under slightly optimized fluence conditions of the proton microbeam, as detailed hereafter. Indeed, as stated in the literature [35], the fluence parameter influences the etching rate and, consequently, the total protocol time.

**Figure 2:**
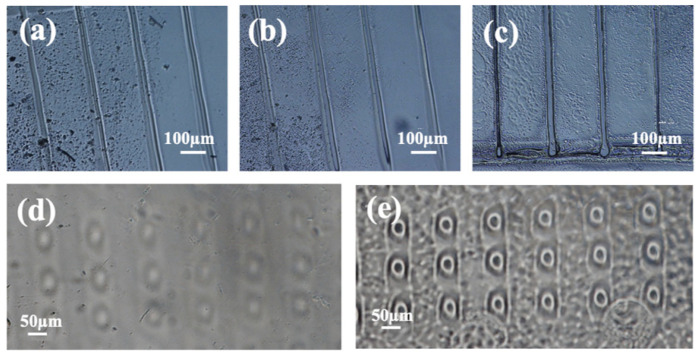
Optical images of the proton microbeam written PDMS channels on (**a**) bulk PDMS before wet etching and after wet etching in a NaOH solution (30% wt) at T = (80 ± 5) °C and incubation times of (**b**) 30 and (**c**) 90 min. (**d**,**e**) Optical microscopy images of the proton beam written holes on a 10 µm-thick PDMS layer on glass (**d**) before wet etching and (**e**) after wet etching in a NaOH solution at (80 ± 5) °C for 90 min, after delamination from the glass substrate and integration into the device, as described in experimental section (see scheme in Figure 1).

Figure 2 shows optical images of the test PDMS channels before the wet etching processing (Figure 2a) and following their immersion in a NaOH (30% wt%) solution at (80 ± 5) °C and at different exposure times (Figure 2b,c). Several preliminary trials demonstrated effective chemical removal of the damaged PDMS for an incubation time of at least 30 min (Figure 2b). Increasing the exposure time was found to deepen the etched channel, leading to the complete removal of the modified PDMS. Indeed, monitoring of the etched depth by a profilometer demonstrated gradual progressive removal of the damaged PDMS for an etching time of up to 90 min. No further relevant deepening was observable for longer immersion times, meaning the underlying undamaged PDMS material was reached (see Figure S2). Scanning Electron Microscopy (SEM) images in Figure S5 also confirm the trend observed by optical microscopy.

On turning from lines to holes, Figure 2d shows optical microscopy imaging of an array of holes, with a nominal diameter of 25 µm and periodicity of 100 µm, written by the proton microbeam before processing by the optimized wet etching protocol. The holes were patterned on a 10 µm-thick PDMS layer deposited on a glass substrate coated with a thin water-soluble sacrificial layer to be removed according to the procedure described in the experimental section for assembling the transwell-like device under study in this paper (see Figure 1). An image of the etched membrane after delamination from the glass support and integration into the device is shown in Figure 2e. For the sake of brevity, the PDMS membrane processed by PBW and drilled by above detailed wet etching protocol will be referred to as “PBW-holed membrane” hereafter.

The PBW-holed membrane shown in Figure 2d appears rougher than the unirradiated PDMS surface shown in Figure 2e due to the sacrificial layer that molds the PDMS layer on the membrane side that was in contact with the glass substrate before delamination (see Figure 1).

The irradiation conditions optimized to obtain 10 µm-deep holes etchable using the same conditions as the ones set-up to etch channels required higher fluences compared to channels of similar width and depth, that is, a current of 60 pA and fluence of 122,293 pC/mm^2^ (corresponding to 7.6 × 10^11^ ions/mm^2^). As a matter of fact, a different etching effect can occur inside the same structure due to the overlapping areas of the beam spot, while it is moved to write the designed shape [35]. Operatively, the lines were defined by the aperture of the collimation slits with defocused quadrupole lenses, thus distributing the charge more homogeneously at the expense of the fluence [37,58]. As the facility used for the PBW method can reach a proton microbeam spot of ~1.2 µm at its best focalization conditions, after the current rise from 6 pA to 60 pA, the triplet of quadrupole lenses (OM150) was slightly defocused to simply achieve a beam spot size of 25 µm in diameter, with the consequent increase in the fluence. Further considerations about the parameters to define the fluence conditions are given in Section S1 of the Supplementary Materials.

Given the complex interplay between the parameters of the PBW technique and the choice of the etching conditions, our experimental outcomes are not straightforward. We believe that the conceived and developed experimental procedure is advantageous for the fabrication of micro-structured thin layers of PDMS integrable into microfluidic devices for several reasons. Indeed, under our experimental conditions, the thickness of the PDMS test layer is much thinner than the penetration depth of a 3 MeV proton microbeam (Figure S3 of Supplementary Materials) [35], meaning no relevant energy loss and lateral scattering are suffered from protons while going through the PDMS layer. This results in drawing patterns along the whole depth of the PDMS layers with microscale lateral resolution. On the other hand, the calibration of the PBW parameters to draw holes and etch them by using the same chemical etching method set-up for lines enables flexible fabrication of mixed-geometry micro-patterns onto the same PDMS layer. For instance, both lines and holes are interesting patterns for microfluidic applications; lines may generate microchannels to allow controlled fluid flow in microreactors [58,59,60], while holes can be useful to design regular patterns of pores in membranes easily integrable into OoC devices [23,61].

For the current application, the combined PBW-wet etching protocol enables the fabrication of a monolithic transwell-like chip in PDMS with reduced number and impact of the fabrication steps. Holes in PDMS membranes are ordered with defined periodicity and size; this is advantageous over commercial membranes, as it allows more flexible design of the geometric constraints (size, arrangement, ordering of the pores) and investigation of cell migration depending on the cell density and aggregation.

### 3.2. Cell Growth, Arrangement, and Migration Across the Designed Transwell-like Devices

After optimization of the holed membrane fabrication parameters, the transwell-like device of interest in this study was assembled (see Section 2.3). The device was placed on a slide, and HCMEC/D3 were seeded on the upper side of the membrane and cultured for 24 h. Atomic Force Microscopy (AFM) measurements demonstrated a roughness of (2.20 ± 0.76) nm for the seeding side of the PDMS membrane (Figure S6). Such roughness characteristics are expected to affect in no way the cell attachment, growth, and proliferation [62].

In order to confirm the successful issue of the PBW technique and of the designed etching protocol (immersion in NaOH 30% wt for 90 min at T = (80 ± 5) °C), the cell migration through the membrane and the impact of changing the interpore distance of the PBW-holed membrane were ascertained. Imaging the adhesion of cells on the bottom coverslip (Figure S7) confirmed that the PBW-holed membrane allowed the cell migration between the two compartments of the device.

**Figure 3:**
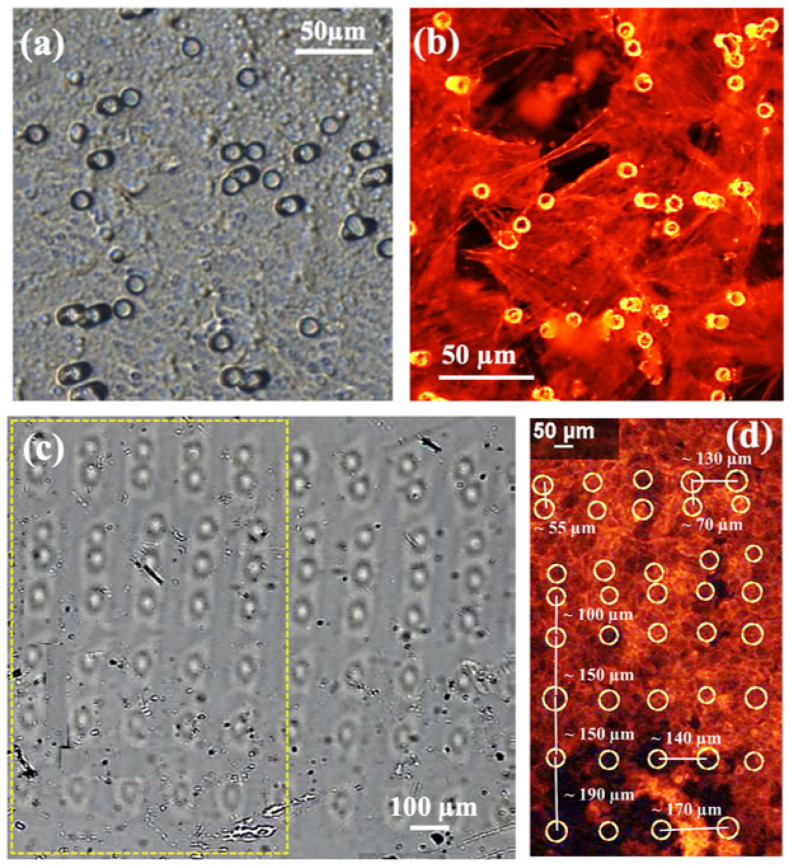
Microscopic images of (**a**) a PC membrane with 8 µm pore diameter and (**c**) an area patterned with the PBW-written hole array; the interhole spacing was varied along two perpendicular directions. The yellow rectangle highlights the representative region of interest shown in panel (**d**). Microscopic images of the phalloidin-TRITC stained cells showing actin filaments of HCMEC/D3 cultured on devices integrating (**b**) a PC membrane with 8 µm pore diameter and (**d**) a PBW-written holed membrane with 25 µm pore diameter and variable interpore spacing along the vertical and horizontal directions. The values reported in image (**d**) indicate the center-to-center interpore spacings. The yellow circles in panel (**d**) highlight the pore positions that, instead, are clearly visible in the case of the commercial PC membrane (**b**).

Figure 3 reports light microscopy images of the cells stained with phalloidin-TRITC showing the actin filaments of HCMEC/D3 cultured on devices integrating a PC membrane with a pore diameter of 8 µm (Figure 3a,b) and a PBW-holed membrane with a pore diameter of 25 µm and varying interpore spacing (Figure 3c,d). Noteworthy, differences in local contrast and gray intensities of the pores between Figure 2e and Figure 3c are due to the fact that while the former case reports the effect of wet etching on the pores, the latter case refers to the spatial arrangement of the PBW-drilled pores before etching and integration in the device.

In the case of the PBW-holed membrane, the circles highlight the positions of the pores, and the values reported on the image were calculated as the center-to-center interpore spacing along the vertical and horizontal directions. Pores are clearly visible in the case of the PC commercial membrane.

The comparison in Figure 3 clearly shows that the transition from a random to a regular distribution of pores induces more uniform proliferation and spreading of the cells. While extended empty regions and localized agglomeration, mainly around the 8 μm diameter pores, are evident in the case of the PC membrane, a more uniform distribution of the cells is clearly allowed by the PBW-holed membrane and, as a confirmation of the properly designed pore size, cells arrange in between the pores as well as around the area to the pore center. When the interpore spacing increases, the cell distribution becomes less homogeneous, cell density decreases and depletion regions form. Therefore, a regular array of properly closely spaced pores provides a higher level of cellular migratory activities and metabolism, as expected due to a uniform uptake of oxygen and nutrients.

The comparison in Figure 3 provides useful guidelines. That is, over the studied pore-to-pore spacing and for the endothelial cell species under study, surface cell growth is optimized for pore size comparable to the cell characteristic length (the average diameter of endothelial cells is 10–40 µm) and interpore distance ranging from 55 µm to 150 µm. These conclusions, which are in agreement with the literature [38], demonstrate the flexibility of the PBW-based design for manufacturing stable micro-porous membranes in a reproducible and engineerable way.

#### 3.2.1. Impact of the Material Membrane on the Arrangement of HCMEC/D3 Cells

In order to assess and characterize the influence of the designed PDMS PBW-holed membrane on the cell arrangement, we investigated by optical microscopy the cellular behavior on the device of interest in comparison to two transwell-like devices integrating a PC membrane and a glass coverslip, respectively.

PC membranes are porous membranes with a pore size of 8 μm in diameter, commonly used in commercial transwell systems to investigate cellular transmigration phenomena (optical image shown in Figure S8a), while glass coverslips are non-porous substrates traditionally used for standard 2D cell culture. Devices integrating such substrates are used as reference controls to compare to the transwell-like device incorporating a PBW-holed PDMS membranes (Figure S8b), whose 25 µm pore size was selected to match the average diameter of endothelial cells (10–40 µm) and has previously been reported to promote cell migration [38]. Despite the different pore size, the investigated PC and PBW-holed membranes are comparable in terms of overall porosity; porosity values of 3.4% for the commercial PC membrane versus 5.4% for the PBW-holed PDMS membrane were calculated using the fractional volume method [63].

**Figure 4:**
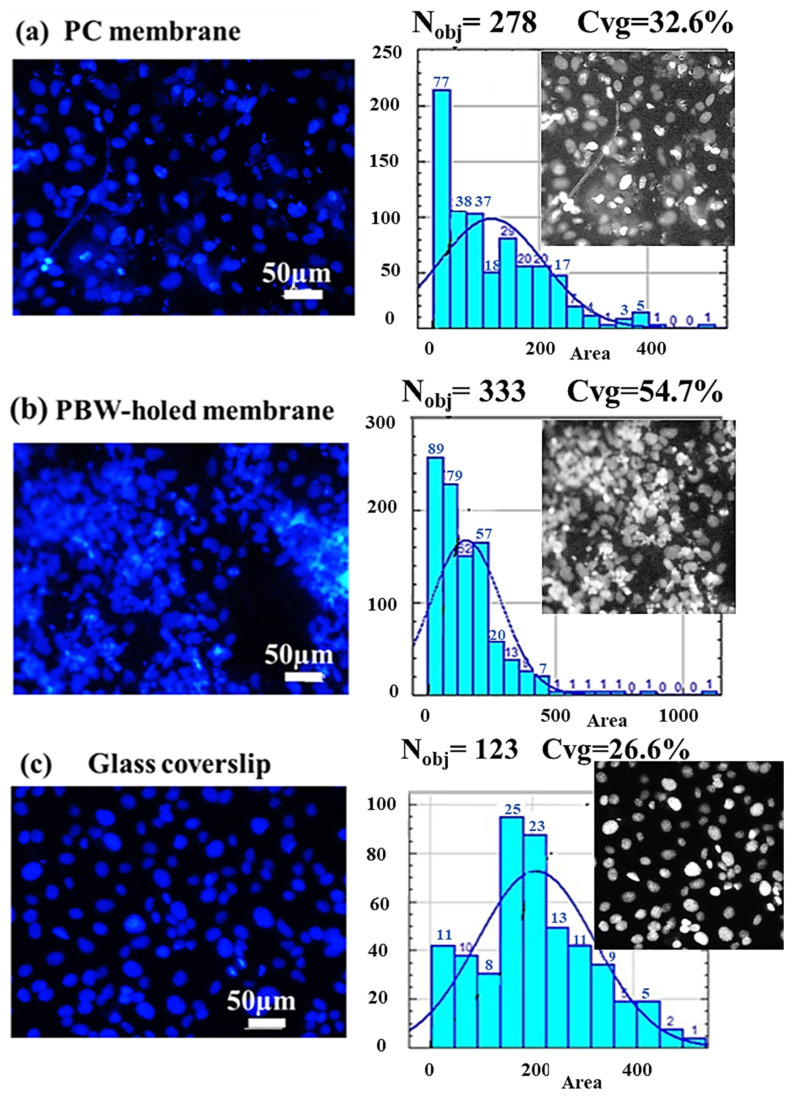
Microscopy imaging of the distribution of HCMEC/D3 cultured on devices integrating (**a**) a PC membrane, (**b**) the PBW-holed membrane, and (**c**) a commercial glass coverslip by the DAPI-channel. The statistical distributions of the area of the nuclei (histograms counting the frequency of the area values), count of objects (nuclei), and the percentage coverage are reported in any case. A ROI with an area of 350 µm × 350 µm is shown as an inset in the panel of the histogram (gray-scale image). This ROI is representative of the nuclei distribution of the sample under consideration.

Figure 4 shows the arrangement and statistics of the blue DAPI-stained nuclei following cell growth onto the three substrates integrated in the devices. The count of the objects (N_obj_) and the percentage value of the coverage (Cvg) are reported in the panel showing the histogram of the distribution of the area of the nuclei (that is, the number of occurrences of each value of area reported on the vertical axis, where the area refer to the area of each nucleus classified in the image).

For the PC membrane (Figure 4a) and PDMS PBW-holed membrane (Figure 4b) devices, statistical analysis and object counting was performed over multiple regions of interest (ROIs) with an area of 350 µm × 350 µm. In particular, a set of two images for each sample and two ROIs per each image were processed. Figure 4 shows representative images of the nuclei and one associated ROI as the inset of the histogram plots. The ROI was selected in such a way that cell-depleted regions, that is, pores, were avoided to allow a reliable comparison in terms of statistics. It can be clearly observed that a larger density of cells proliferates over the PBW-holed membrane, despite the larger size of pores that would be expected to favor a larger rate of cell migration across the membrane. On a rigid substrate, such as the glass coverslip, cells cover the entire surface showing clearly nuclei more dispersed than in the case of the holed membranes (see Figure 4c). The gray-level distribution reported by the ROI images indicates a trend to cell clustering and 3D proliferation of the cells in the case of the drilled membranes. This trend is much more evident for the PBW-holed membrane. A 2D sparse distribution can be observed in the case of the glass coverslip support.

Differences in the arrangement of HCMEC/D3 on the substrates under examination were also confirmed by imaging the actin filaments via the phalloidin-TRITC channel.

**Figure 5:**
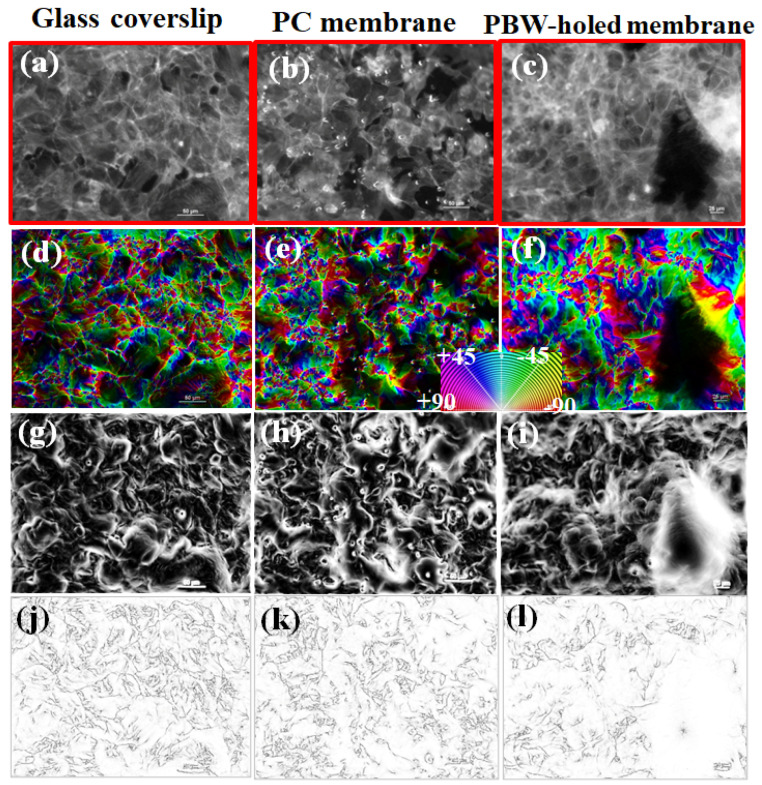
HCMEC/D3 on devices integrating glass coverslip, PC membrane, and PBW-holed membrane. (**a**–**c**) Images of cells stained with phalloidin-TRITC showing the actin filaments distribution. (**d**–**f**) Orientation spatial maps associated with the input image calculated based on the gradient structure tensor. (**g**–**i**) Energy spatial maps associated with the input images, where energy E is the trace of the gradient structure tensor. (**j**–**l**) Coherence spatial maps calculated based on the tensor-structure operator. The scale bar (white segment) is 50 μm.

**Figure 6:**
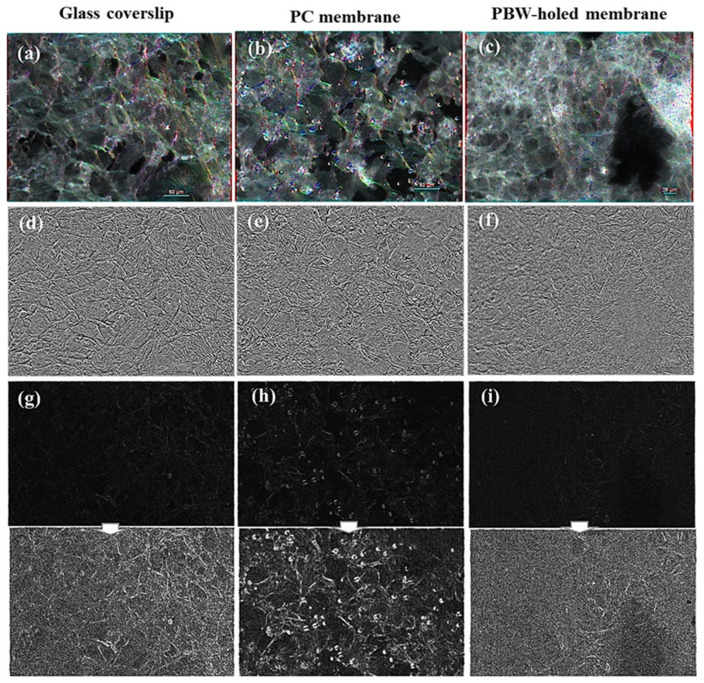
Monogenic signal analysis of the HCMEC/D3 on devices integrating glass coverslip, PC membrane, and PBW-holed membrane. The images reported in Figure 5a–c were processed, that is images of cells stained with phalloidin-TRITC showing the actin filaments distribution. (**a**–**c**) Spatial maps of the monogenic direction associated with the input image. (**d**–**f**) Spatial maps of the monogenic phase. (**g**–**i**) Monogenic modulus images with the corresponding contrast-enhanced images indicated by white arrows. The scale bar (white segment) in panels (**a**–**c**) is 50 μm.

Going beyond the visual inspection of the acquired fluorescent microscopy images of the phalloidin-TRITC red channel, the geometrical structure features of the cell distribution corresponding to the nuclei mapped in Figure 4 were investigated by the gradient structure tensor approach (Figure 5) and the monogenic analysis (Figure 6) applied to the phalloidin-TRITC red channel associated with the cell arrangement on the glass coverslip, PC membrane, and PBW-holed membrane, respectively (Figure 5a–c).

Figure 5d–f and Figure 6a–c report on the orientation spatial maps calculated by combining the gradient structure tensor with the Riesz filter (Figure 5) and the monogenic representation of the input image (Figure 6). In this respect, orientation is encoded in the HUE color appearance (hue–saturation–brightness map, where HUE is associated with the orientation, saturation is coherency, and brightness is the original fluorescence image). The color-orientation wheel is reported. Figure 5g–i show the energy spatial maps associated with the input images, where energy is defined as the trace of the gradient structure tensor. The distributions of white-color regions (high energy pixels) and black-color regions (low energy pixels) enable us to visualize the occurrence of locally oriented features marking the directions of relevant changes in the gradient tensor within the source image (see the Section 2.5, point (i)). Figure 5j–l report the coherence spatial maps, where white regions are associated with anisotropy. The information provided by Figure 5 is that the cell arrangement exhibits a distribution of local orientations and high anisotropy for any substrate under examination (glass coverslip, PC membrane, and PBW-holed membrane).

The energy spatial maps of all the samples show the occurrence of white features (high-energy pixels) with curl-like geometry that are indicative of large variation in the gradient direction correlated with the cellular arrangements.

Turning to the monogenic analysis shown in Figure 6, orientation (panels (a–c)), phase (panels (d–f)), and modulus (panels (g–i)) are shown. The monogenic modulus maps are very low contrast images with very weak maxima. In order to improve the visibility of these maxima, a contrast enhancement post-processing was applied. The resulting images, which are indicated by white arrows, enable us to associate the maxima of the monogenic modulus to the objects occurring in the image. Compared to orientation and phase, the monogenic modulus is the less informative quantity. The orientation maps in panels (a–c) confirm the occurrence of local orientations in the fluorescence images and enable us to associate the occurring orientations with the cell filaments in a more direct fashion with respect to the gradient tensor. The monogenic phase maps reported in Figure 6d–f allow us to highlight substrate-dependent morphological differences in the cell arrangement and information not provided by the gradient tensor analysis. Indeed, the ability of the phase to detect corners and edges lets mark the occurrence of elongated and sparse cell filaments and mark differences in the geometrical structure of the cell arrangement between the samples under examination that are not so evident based on the gradient energy and coherence maps in Figure 5. In the case of the glass coverslip, elongated and sparse actin filaments can be observed that turn to higher density and shorter filaments in the case of the PBW-holed membrane.

In conclusion, the structure tensor (Figure 5) and monogenic signal (Figure 6) image processing approaches allowed us to point out supporting membrane-dependent differences in the distribution of the actin filaments in terms of main variation direction, orientation map, anisotropy/isotropy, and detection of the geometrical structure. Given a traditional thresholding of the images showing the red-stained actin filaments is prohibitive, texture analysis can be helpful to characterize differences, for instance roughness or bumpiness, among the images as a function of the spatial variation in pixel intensities correlated with the cellular arrangements. Parallelly, the monogenic analysis demonstrates that cells distribute according to local domains with preferential orientations and their elongation is affected by the substrate in terms of changes in the cell density and curvature. This conclusion suggests a further investigation in the impact of the membrane nature in terms of short-range cell arrangement and anisotropy.

**Figure 7:**
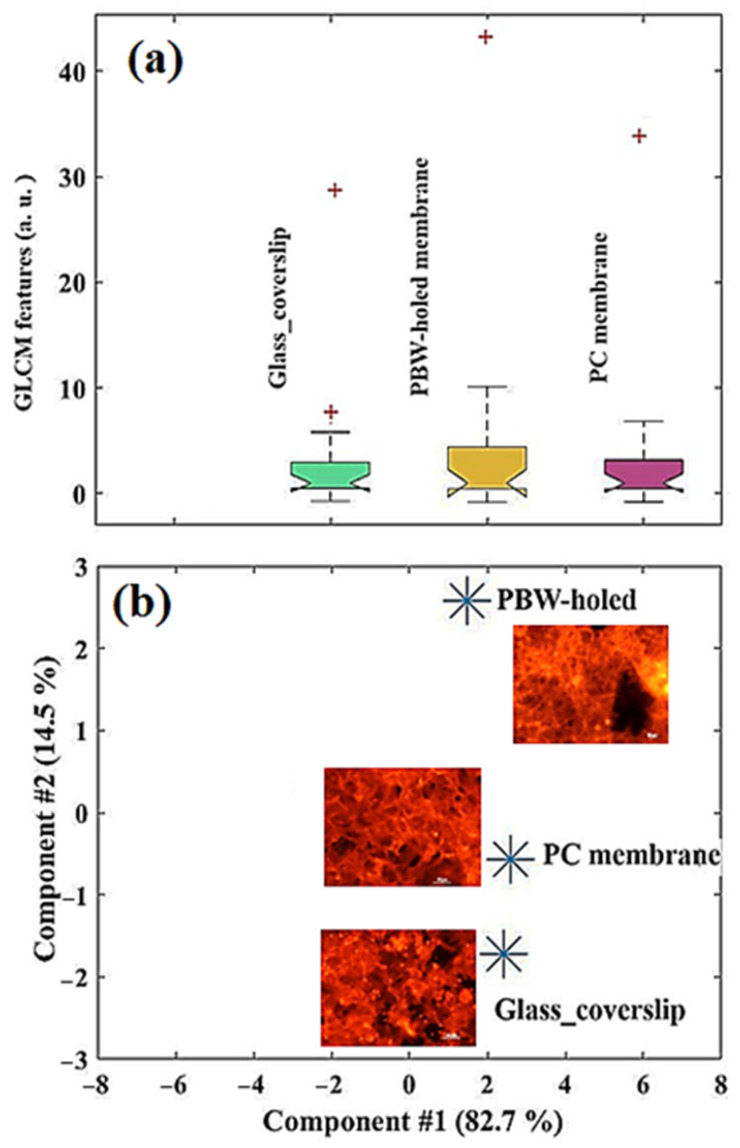
(**a**) Distribution of the GLCM features associated with the phalloidin-TRITC channel of the samples of interest (glass coverslip, PBW-holed membrane, and PC membrane). (**b**) Plot of the first two PCA components calculated for the images reported closely to the corresponding point in the PCA two-component space.

In order to go beyond qualitative visual perception of texture, feature extraction was performed by processing the phalloidin-TRITC source images of the samples of interest by means of the GLCM, and a standardization (mean = 0 and variance = 1) of all the GLCM features was calculated. Technical details were described in the section devoted to methods. To better appreciate the distribution of the features in each case, a box plot was drawn (Figure 7a) [64]. On each box, the central mark indicates the median, and the bottom and top edges of the box indicate the 25th and 75th percentiles, respectively. The whiskers extend to the most extreme data points not considered outliers, and the outliers are plotted individually using the ‘+’ symbol. The GLCM distributions are rather close to each other, with few outliers, more important in values for the PBW-holed membrane and the PC membrane.

Since, PCA is quite sensitive to the variances of the initial variables, a standardization (mean = 0 and variance = 1) of all the GLCM features was performed. Figure 7b shows the distribution of points in the principal component space; the first and second component retain 82.7% and 14.5% of information, respectively, i.e., over 97% of information in total. This means that the discussion of these two PCA components is significant.

The PCA two-component map enables us to observe that the GLCM features associated with the phalloidin-TRITC channel of the glass coverslip and PC membrane could be clustered, meaning they exhibit quite similar texture. More evident differences can be found when comparing the PBW-holed membrane to the PC membrane. The GLCM-PCA analysis showed that the GLCM features for the glass coverslip and PC membrane clustered together, indicating their similar texture as expected, whereas the PBW-holed membrane showed distinct textural differences.

**Figure 8:**
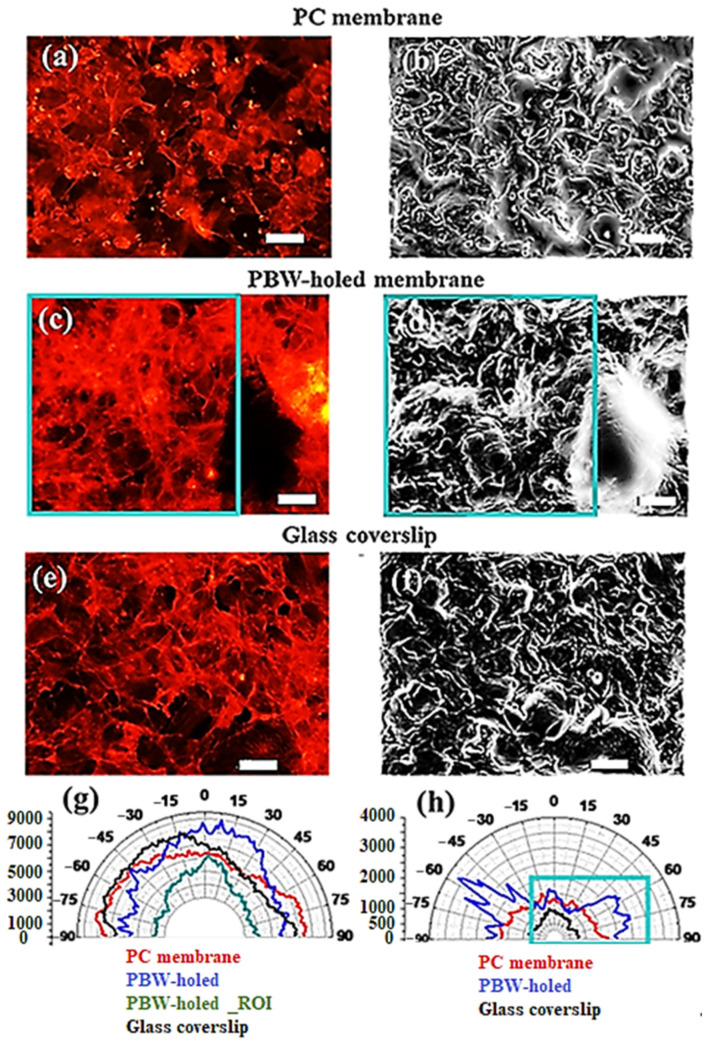
HCMEC/D3 on devices integrating PC membrane, PBW-holed membrane, and glass coverslip: (**a**,**c**,**e**) images of cells stained with phalloidin-TRITC showing the actin filaments distribution, (**b**,**d**,**f**) energy maps calculated based on the tensor-structure operator. (**g**) Polar plots of the distribution of orientations for a commercial PC-membrane (red line), the glass coverslip (black line), the PBW-holed membrane (blue line), and its ROI in the hole-free area (green line). (**h**) Polar plots of the distribution of orientations associated with the white features in the energy maps shown by the panels (**b**,**d**,**f**). The green rectangle points out the contribution of the hole-free ROI (green rectangles superposed to the energy map in panel (**d**)). The scale bar (white segment) is 50 μm.

In order to gain deeper insight in the occurrence or local ordering in the cell arrangements, an unforeseen analysis approach was developed consisting of studying the orientational distribution of the main anisotropy features rather than the orientational distribution. Since the energy associated with the gradient structure tensor indicates/maps the features responsible for the major variation and anisotropy in the image, the orientational distribution of the white features occurring in the energy spatial distribution was investigated. In other words, a polar plot of the orientations of the white-color energy anisotropy characteristics of the image was graphed to disclose differences among the membranes in terms of preferential anisotropy directions of the seeded cells. In this respect, Figure 8a,c,e shows fluorescent microscopy images of the phalloidin-TRITC red channel of the cell distribution on the top of the randomly distributed pores of the PC membrane, of the ordered pores of the PBW-holed membrane, and of the glass coverslip (corresponding to the nuclei mapping of Figure 3), respectively. The gradient energy maps reported in Figure 8b–f are associated with the images in panels (a–e) and were obtained by processing the source images of the phalloidin-TRITC channel over their entire area by means of OrientationJ.

Figure 8c,d shows the actin distribution on the PBW-holed membrane with a wide area depleted by cells, that is, well visualized by the large and intense white curl-like structure of the energy map. Figure 8e,f represent cell coverage on the glass coverslip with a homogenous distribution and isotropy/anisotropy conditions more similar to the PC membrane rather than to the PBW-holed membrane.

The distributions of the orientation of the main variation features reflecting the distribution of the actin filaments in the source images were graphed using the polar plot representation as reported in Figure 8g, where angles refer to orientations in the plane of the images. The drawn polar plots reveal that cells on the glass coverslip (black line) are oriented within a quite large band that exhibits a weak asymmetry due to a preferential orientation around—45°. A similar wide band can also be observed in the case of the PC membrane (red curve), meaning no evident preferential orientation. Regarding the cell orientation on the PBW-holed membrane, the polar plot on the entire area of the source image (blue curve) shows evident differences with respect to the other cases; in particular, a quite symmetric distribution of orientations is evident. Investigation conducted over a ROI restricted to the hole-free area (green curve) indicates that cells preferentially oriented along the 0° direction. Therefore, as a matter of fact, the polar plot calculated over the entire image includes sub-orientations associated with colonized sub-domains. These conclusions are also supported by the polar plot in Figure 8h, which reports the analysis of the anisotropy main orientations associated with the white high-energy features occurring in the energy maps of Figure 8b,d,f.

On turning from the orientational distribution of HCMEC/D3 to the orientational distribution of the associated anisotropy features, the differences between the samples under consideration are more evident. It can be observed that PC membrane and glass coverslip exhibit a contribution from widely distributed orientations, accordingly with the information provided by the polar plot in Figure 8g. In the case of the PBW-holed membrane, the energy map points out the occurrence of different cell orientations more clearly; an ROI (green rectangle) avoiding the depleted region around the single hole includes several specific orientations, as highlighted by the green rectangle in Figure 8h that encloses the anisotropy orientations stemming from the hole-free ROI.

In conclusion, the PC membrane and glass coverslip both displayed a similar, more homogeneous cell coverage with fewer empty regions compared to the PDMS PBW-holed membrane. However, on the glass coverslip, endothelial cells were more planar and less clustered, forming orientations influenced by the alignment of actin filaments due to strong cell–cell interactions. This behavior is attributed to the cell tendency to align actin filaments in preferred directions on flat and rigid substrates, where the cells experience uniform mechanical forces. This mechano-transduction process involves the cells converting mechanical signals into biochemical signals, leading to changes in cytoskeletal organization, which stabilizes and compacts the cell layer [65]. In contrast, this type of cell organization is less evident in the suspended and porous membrane systems, both PC and PDMS-based membranes. The inherent flexibility of these porous membranes, combined with their porous architecture, promotes 3D clustering and perturbs the self-organization commonly observed over unfeatured continuous areas. These observations are essential for studying the endothelial cell behavior in the context of transmigration and angiogenesis. This is especially true for HCMEC/D3 cells, a well-established human brain microvascular endothelial cell model widely used to study vascular maturation in the brain [29,61]. These cells are of particular interest for modeling the development of the blood-brain barrier (BBB), where microvascular functionality emerges through intricate cellular interactions during central nervous system angiogenesis.

Understanding how endothelial cells respond to different mechanical and structural cues sheds light on their capacity to migrate, invade, and form organized vascular structures—key events in both physiological (e.g., tissue repair) and pathological (e.g., tumor vascularization) angiogenesis [21,36,66]. Therefore, the gained morphological insights, enabled by advanced image processing approaches applied herein, provide a robust foundation for future investigations into endothelial cell behavior during barrier remodeling and transmigration across membranes. This knowledge will be crucial for the development of effectively predictive in vitro models for vascular biology and drug screening.

#### 3.2.2. Deeper Insight in Cell Arrangement Around and in Between the PBW Holes

**Figure 9:**
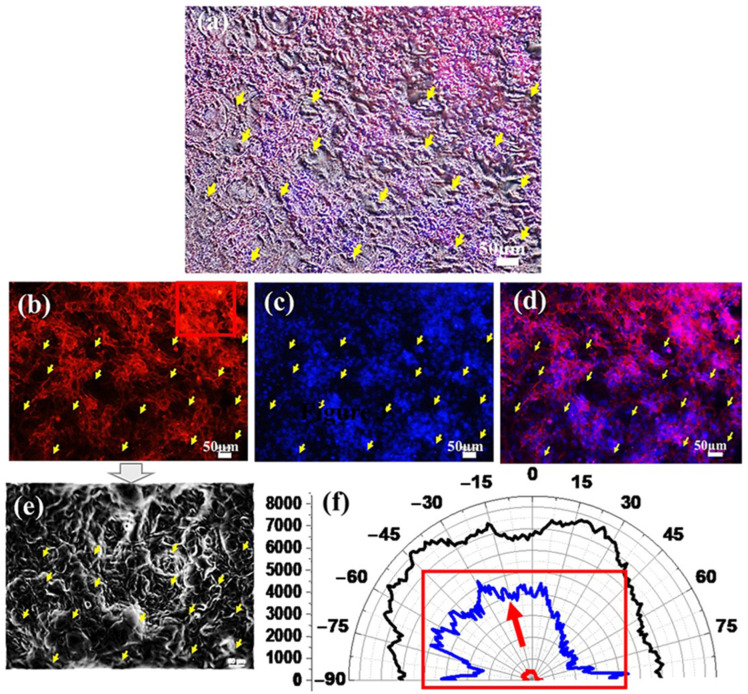
Light microscopy imaging of HCMEC/D3 on the surface of a transwell-like device integrating the PBW-holed membrane at different magnification. (**a**) Bright field image merged with phalloidin-TRITC and DAPI channels showing the distribution of the of HCMEC/D3 with the yellow arrows highlighting the placement of the PBW-processed holes. Large area images of (**b**) phalloidin-TRITC channel, (**c**) DAPI channel, (**d**) overlap of phalloidin-TRITC and DAPI channels. (**e**) Energy spatial map of the phalloidin-TRITC channel in panel (**b**). (**f**) Polar plot of the orientations of the actin filaments over the area of the shown phalloidin-TRITC channel (black curve) and over a ROI pointed out by a red square in panel (**b**). The red and green polar plot curves are the distribution of orientations associated with the ROI and its multiplication by 10.

Figure 8a, obtained as an overlap between bright-field optical and fluorescence channels, reports several situations—depleted regions around a hole and cell accumulation over a hole preliminary to cell migration across the hole, as expected during the occurrence of transmigration processes. Representative images over a large area are also reported (Figure 9), allowing us to observe the ordered distributions of PBW-induced holes and the cell arrangement in between and around them.

The yellow arrows in Figure 9a allow us to locate the PBW-processed holes and their relative placement with respect to the cellular features of interest (actin filaments in the phalloidin-TRITC channel (Figure 9b) and nuclei in the DAPI channel (Figure 9c). The merged phalloidin-TRITC channel plus DAPI channel is also shown (Figure 8e). The dark regions are consistent with depleted areas associated with the occurrence of pores. Cells are unable to form a confluent monolayer over the imaged surface due to the occurrence of the pores (Figure 9a) that work effectively in driving cell migration from the topmost to the bottom surface of the membrane (Figure S7). The monogenic signal analysis of the images in Figure 9a and over an area close to a pore are shown in Figures S8 and S9 of the Supplementary Materials.

The image picturing the phalloidin-TRITC channel was also processed to obtain the energy spatial map (Figure 9e,f). Yellow arrows in Figure 9e, corresponding to the spatial location of the pores in the microscopy images, also correspond to regions with high-energy white features, meaning that major variations in the structure of the image and anisotropy in the cell distribution are introduced by the pores. This evidence is consistent with the discussion related to cell distribution around a single pore.

Orientations mapped over the entire area indicate the occurrence of a broad distribution of orientations (black curve in Figure 9f), including a few preferential directions that can be understood based on the analysis over a ROI restricted to the hole-free area shown in Figure 9b. Given the low signal stemming from the remarkably smaller extension of the sampled ROI, the associated polar plot (red curve) was multiplied by 10 (green curve) to make it clearly comparable to the black curve. Hence, Figure 9f demonstrates cell alignment along a specific direction, meaning that unperturbed high-density cells have actin filaments strictly aligned with each other locally. This conclusion was ascertained by a systematic analysis performed over multiple ROIs randomly selected over the surface of a large-area microscopy image of the PBW-holed membrane (see Figure S10 of Supplementary Materials).

In conclusion, when the membrane pores are comparable in size to the adhering cells (as in the case of the PC membrane), it is difficult to identify large empty areas. Differently, larger pores, such as in the PBW-holed membrane, not only favor depletion regions but also enable a richer set of cell arrangements. Indeed, this emptying effect creates regions near the pores where the cell layer modifies, showing preferred directions in small areas (as seen in Figure 8g, green plot, and Figure 9f, blue plot). These areas are likely where the clustered, 3D cell multilayer thins out and distributes over smaller domains, leading to a reorganization based on more ordered cell–cell interactions, resulting in the well-defined actin filament orientations observed in polar plots.

### 3.3. Remarks and Future Perspectives

In this study, we developed a PBW-based fabrication method to produce porous membranes and advanced image processing approaches to point out differences in the distribution of HCMEC/D3 depending on the nature of substrate. Several interesting discussion points raised questions, and future perspectives can be listed based on the developed discussion and outcomes.

(i) The PBW-holed membrane demonstrated in this study is a prototypical example that does not limit the generality and potential of the PBW technique in terms of fabricating porous membranes with customizable pore shape and size, membrane thickness, and interpore spacing. Indeed, structures with different geometrical shapes and relative spacing as well as complex arrays on the same support can be obtained by a properly designed scanning of the irradiated area. In our experimental conditions, although proton beams can be focused down to a 1.2 µm × 1.2 µm area of the beam cross-section, in order to customize the devices to study interactions of the endothelial cells with pores and their migration tendency, pores with consistent dimensions and interpore spacing were fabricated.

(ii) The developed analysis underscores the profound impact of transitioning from rigid, planar substrates to porous, flexible ones on cellular self-organization and mechano-transduction processes. In particular, HCMEC/D3 exhibited distinctive migratory behavior when grown near the 25 µm diameter PBW pores of the PDMS membrane, activating migratory patterns associated with cytoskeletal remodeling, which is a critical element in the angiogenic process [21,36,67]. Migration of endothelial cells is a dynamic and complex mechanism governed by signaling pathways converging on cytoskeletal reorganization, which is essential for initiating angiogenic sprouting [21,36,67]. Our findings provide preliminary insights into the mechanisms underlying HCMEC/D3 migration, with a particular focus on cell distribution and alignment influenced by the porous structure of the PDMS PBW-holed membrane. While previous works reported the development of 3D angiogenesis models on chip that, despite their innovativeness and biological fidelity, could not highlight the migratory processes that precede and promote sprouting due to the system’s architecture [67,68], our study shows a method for the fabrication and analysis of in vitro systems, enabling a more predictive modelling of migration phenomena underlying angiogenic events.

(iii) The physical properties of a substrate—such as rigidity, porosity, and texture—play a critical role in shaping cell behavior, influencing key processes like arrangement, migration, orientation, and morphology [69,70,71]. In this regard, it is worth underlining that these insights pave the way for new explorations into how substrate characteristics can be tailored to modulate cellular responses effectively. Pore size, in particular, has been identified as a critical factor in promoting angiogenesis within vascular interfaces of implanted biomaterials [68,72]. However, angiogenic functionality is also influenced by other material properties, including membrane thickness, pore interconnectivity, and surface treatments [21]. Future studies integrating pore size with other material properties, such as membrane thickness, pore density, and surface modifications, could further optimize substrates for enhanced angiogenesis and improved integration within in vitro models.

## 4. Conclusions

This work discusses a method to conveniently study processes of cellular organization through the design and realization of a monolith PDMS transwell-like device. Its performance is demonstrated by combining light microscopy imaging with advanced image processing approaches (GLCM texture analysis, gradient structure tensor, and monogenic signal) that allow us to go beyond standard visual inspection and conventional statistics as well as to overcome the challenging segmentation of complex images. It is worth observing that we used publicly available tools (FiJi/ImageJ) and that the set-up reasoning can be generally applied to study the distribution of any biological species over any type of support.

The membrane of the transwell-like device was realized according to the following two main steps: in step 1, PBW was implemented to transfer a designed pattern over a polymeric material through local physico-chemical modification of the irradiated area, and in step 2, chemical etching allowed us to remove the regions modified by the polymer–proton microbeam interaction.

From a fabrication perspective, the procedure proposed herein has the remarkable advantage of the reduced number of fabrication steps and of increased design flexibility, compared to methods fully based on optical lithography and wet etching. Membranes with any micro-sized diameter of the pores and pore geometry as well as with regular pore size and distribution can be obtained, with advantages of greater experimental reproducibility and easier visualization compared to commercial membranes.

The designed transwell-like device proved to be particularly suitable for studies on cellular organization. This aspect was demonstrated by the seeding of endothelial cells on devices that incorporated PBW-holed membranes with different interpore distances, particularly interesting for angiogenetic studies, and by imaging the cell arrangement by fluorescent microscopy. Thanks to the high control of PBW manufacturing method, the impact of the interpore distances on the cellular depletion around the pores was evaluated.

Cell distribution and orientation were also discussed in comparison to transwell-like devices integrating commercial membranes (PC membranes) and rigid standard substrates (glass coverslip). Pores in the PBW-holed membranes acted as effective depletion regions around which cells converge and appeared more oriented, as an effect of local cell-to-cell rearrangement induced by cell migration. Hence, cell orientational short-range ordering was observed in the area in between the pores, despite the high tendency of clustering on flexible PDMS membranes. Similar behavior was not observed in the case of PC membranes where cell transmigration occurred without leading to large regions of depletion. The observed membrane-dependent behavior is due to the relationship between pores size and cell size, as described in the literature [38].

The comparison between devices incorporating substrates of different stiffness and porosity offered the possibility to discuss the importance of mechano-transduction processes with important insights in the field of in vitro modelling, biomaterials engineering, and vascular research. Indeed, the type of device and the proposed advanced optical image processing can represent a very interesting basis for creating and studying in vitro systems, such as OoCs designed to mimic the functional characteristics of organs, and allow them to be studied in physiological or pathological conditions.

## Data Availability

The original contributions presented in the study are included in the article, further inquiries can be directed to the corresponding author.

## Supplementary Materials

The following supporting information can be downloaded at https://www.mdpi.com/article/10.3390/jfb16080274/s1. Figure S1. Scheme of the different pattern developments when applying the PBW method; Figure S2. Chemical structure of polydimethylsiloxane (PDMS); Figure S3. SRIM simulation performed on a PDMS target showing an overall 3 MeV proton beam penetration of 180 μm; Figure S4. Profilometric analysis of microchannels during wet etching in NaOH at increasing incubation times; Figure S5. (a,b) Optical images of the proton beam written PDMS channels on bulk PDMS (a) before wet etching and (b) after wet etching in a NaOH solution (30% wt) at T = (80 ± 5) °C and incubation times of 90 min. (c,d) Scanning Electron Microscopy images of the proton beam written PDMS channels on bulk PDMS after wet etching in a NaOH solution (30% wt) at T = (80 ± 5) °C and incubation times of 90 min; Figure S6. 3D image from an AFM acquisition of the PDMS membrane deposited on top of a PEDOT-coated glass coverslip over an area of 40 µm × 40 µm; Figure S7. HCMEC/D3 cells on the flat glass surface located at the bottom side of the chip including the PBW-holed PDMS membrane. (a) DAPI channel, (b) phalloidin-TRITC channel, (c) merge of phalloidin-TRITC and DAPI channels; Figure S8. (a) Bright field image merged with phalloidin-TRITC and DAPI channels showing the cell arrangement around a PBW-induced hole. (b) Image of cells stained with phalloidin-TRITC showing the actin filaments distribution around a PBW-induced hole. (c,e,g) Spatial maps of the monogenic orientation, phase and modulus associated with the image in panel (a). (d,f,h) Spatial maps of the monogenic orientation, phase and modulus associated with the image in panel (b); Figure S9. (a) Bright field image merged with phalloidin-TRITC and DAPI channels showing the distribution of the HCMEC/D3 over the PBW-processed holes. (b) Image of cells stained with phalloidin-TRITC showing the actin filaments distribution over the PBW-holed membrane. (c,e,g) Spatial maps of the monogenic orientation, phase and modulus associated with the image in panel (a). (d,f,h) Spatial maps of the monogenic orientation, phase and modulus associated with the image in panel (b); Figure S10. HCMEC/D3 cells on devices integrating a PBW-holed membrane. (a) Light microscopy image of cells stained with phalloidin TRITC showing the actin filaments distribution stained in red color. (b) Local analysis tracking the occurrence of preferential orientations of the cells over randomly distributed ROIs. (c) Energy map associated with the image in panel (a) calculated by the tensor- structure operator. (d) Polar plot of the distribution of orientational anisotropy associated with thewhite features in the energy map shown in panel (c). References [35,37,72,73,74,75,76,77,78,79,80,81,82,83,84] are cited in the supplementary materials.

## Abbreviations

The following abbreviations are used in this manuscript:

CEDAD	Centre of Applied Physics, Dating, and Diagnostics
DAPI	4′,6-diamidino-2-phenylindole
GLCM	Gray Level Co-Occurrence Matrix
HUE	Hue–saturation–brightness
HCMEC	Human Cerebral Microvascular Endothelial Cells
OoC	Organ-on-chip
PBS	Phosphate Buffered Saline
PC	Polycarbonate
PDMS	Polydimethylsiloxane
PET	Polyethylene terephthalate
PCA	Principal Component Analysis
PBW	Proton beam writing
ROI	Region of interest
SEM	Scanning Electron Microscopy
TRITC	Tetramethylrhodamine isothiocyanate
3D	Three-dimensional
2D	Two-dimensional
