# Supplementary material for: PDMS Membranes Drilled by Proton Microbeam Writing: A Customizable Platform for the Investigation of Endothelial Cell–Substrate Interactions in Transwell-like Devices
Source: J Funct Biomater. 2025 Jul 28;16(8):274. doi: 10.3390/jfb16080274 (PMC12387213; doi:10.3390/jfb16080274)
Supplement: Supplementary file 1 [file jfb-16-00274-s001.zip › jfb-3746085-supplementary.pdf]

Supplementary Materials

Supplementary Materials section S1: Proton Beam Writing (PBW)

Proton Beam Writing (PBW) based on a MeV-focused proton beam is a powerful method to produce accurate micrometric high-aspect-ratio structures at micrometric and nanometric levels [72,73,74]. Indeed, while interacting with matter, a proton beam can retain a straight-line pathway thanks to its acceleration and the high proton mass if compared with keV electron probes. Consequently, a MeV-focused proton beam suffers very low energy transfer and minimal scattering for each ion-electron collision with a non-significant deviation, deeply interacting with the target material until releasing all its energy at the end of its path (Bragg peak) [75,76].

Unlike sputtering techniques, like Focused Ion Beam (FIB), a proton beam cannot directly eject or erode the irradiated sample. Instead, it induces local chemical changes (as chain scissions or further crosslinks) in such a way the exposed areas become sensitive or resistant to specific solvents, hence allowing selectively developed structures by chemical etching [77,78].

Therefore, depending on the chosen chemical protocol, the sample can be defined as a positive or negative resist when the etchant removes, respectively, the irradiation-induced changes or the unirradiated areas (Figure S1). Consequently, sharp micro-sized patterns can be directly written and then developed without using intermediate molds and masks. All of this is particularly relevant for simplifying and accelerating the production steps of integrated devices and platforms, such as Lab on Chip (LoC) or Organ on Chip (OoC) devices**.**

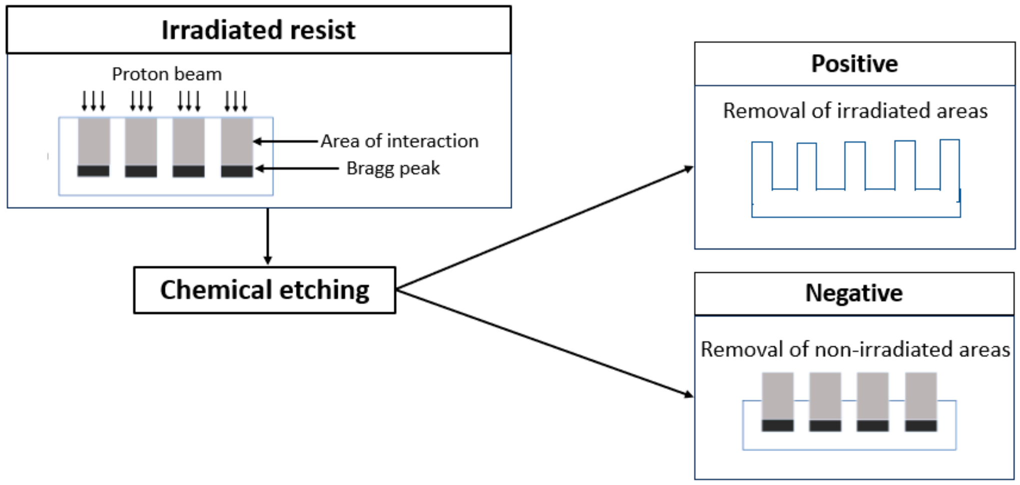

Figure S1. Scheme of the different pattern developments when applying the PBW method.

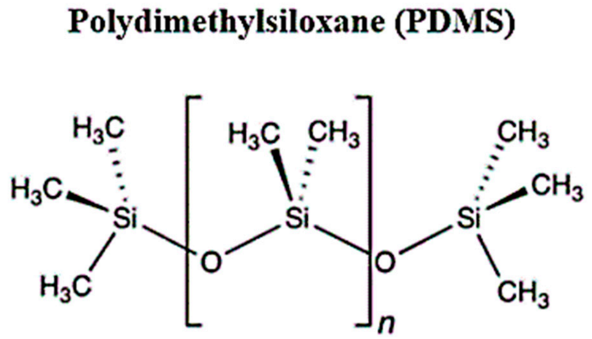

Figure S2. Chemical structure of polydimethylsiloxane (PDMS).

For this study, polydimethylsiloxane (PDMS) (Figure S2) was chosen as a polymeric positive resist on which testing different patterns drawn by PBW applied under different conditions to identify the best operational parameters [35]. Indeed, the current of the microbeam, the proton density and energy can determine the time needed to impress the resist and its structural changes, hence defining the etchant conditions.

A first evaluation of the working parameters for the development of different geometries obtained using a 3 MeV proton microbeam was realized on a PDMS bulk block and a 20 μm thick PDMS layer spinned on a glass slide. The tests on different shape and sizes were necessary to evaluate the optimal fluence conditions and to optimize the chemical etching protocol.

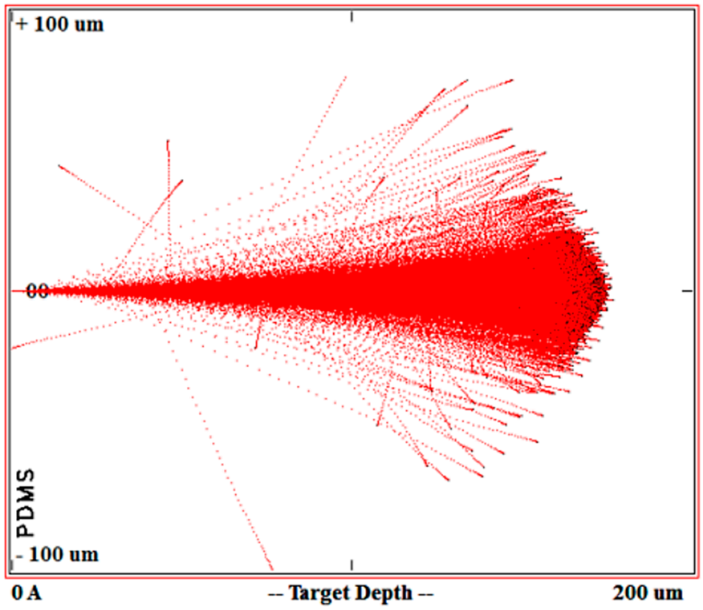

Figure S3. SRIM simulation performed on a PDMS target showing an overall 3 MeV proton beam penetration of 180 μm.

When using such a highly energetic proton microbeam (3MeV), the maximum penetration in PDMS is 180 µm (Figure S3), as found by simulating the possible interaction using *Stopping and Range of Ions in Matter* (SRIM) licence-free software. Consequently, the 20 µm thick PDMS layer is homogeneously irradiated by protons.

Table S1 lists the geometrical patterns written by a 3 MeV proton microbeam and the relative irradiation conditions (current and fluence values) allowed by the used facility. Indeed, the development of an irradiated structure depends not only on the selectivity chemical etching protocol but also on the irradiated areas, the energy of the proton microbeam and the fluence, defined as the number of particles per time unit impacting on the target area. In turn, the properties of the proton microbeam depend on the different sections that make up the facility, which can be chosen in relation to specific or general fields of applications [79]. Therefore, despite the protocol developed by Szilasi and Cserháti [35] the main differences between the two facilities involve the ion source, the particle accelerator and the focalization set-up) [80, 37], so the PBW procedure had to be adapted/calibrated for the present case study, specifically with a sputter ion source (Negative Sputter Ion Source HVEE model 860A), a set of three quadrupole lenses (OM150) and a more energetic microbeam (3 MeV).

Table S1. Different geometries tested on PDMS during the checking for the fluence parameters.

**Geometrical pattern**	**Current**	**Fluence/sec**		**Total fluence**	
		**pC/mm^2**	**ions/mm^2**	**pC/mm^2**	**ions/mm^2**
**Thick lines**	1 nA^{8}	48000^{8}	2.9x10^{11}	48000^{8}	2.9x10^{11}
Thin lines	6 pA^{8}	400^{8}	2.9x10^{9}	6000^{8}	3.7x10^{10}
Spots	1 nA^{8}	2040^{8}	1.2x10^{13}	30600^{8}	1.9x10^{14}

Considering the proton microbeam can irradiate a sample with currents ranging from nA to fA, to reach closer fluence values with respect to the abovementioned quoted protocol, the microbeam current was set at 1 nA for the tests on the spot pattern with 50 µm as the diameter. These conditions let reach a fluence value of the order of 10^{13} ions/mm^2·sec and a total fluence of 10^{14} ions/mm^2·sec for a reasonable time value of 16 sec per spot. When enlarging the area for the realization of thick lines, the proton density decreases despite maintaining the current in the nA order. Reducing the pattern size to write the thin lines (25 µm as the shortest side) with the further adjustment of the collimation slits, the current inevitably drops to pA values due to the reduction of the fraction of the proton beam reaching the microbeam line. The test was performed using 6 pA, with the consequent total fluence of 3.7x10^{14} ions/mm^2·sec. Therefore, in view of producing a PDMS holed membrane (25 µm in the pore diameter), a pA current was demanded.

As a result of the fluence decrease, the chemical etching procedure involved long time etchant exposition, but without preventing the pattern development. So, despite the decreased fluence conditions with respect to the literature [35], the pA current with a 3 MeV proton microbeam was sufficient to etch the pattern using the presented chemical protocol.

Bearing in mind that the brightness of a proton microbeam is defined at the ion source and cannot be increased along its path [81], further adjustment of the collimating slits and the focusing quadrupole lenses led to the increase of the microbeam current up to 60 pA and a fluence of 122293 pC/mm^2·sec, as reported in our study, to ensure the better performance in the etch rate for the newly designed chemical protocol.

Supplementary Materials section S2: Profilometer measurements

To confirm that the 90 min etching time was sufficient to remove the entire PDMS layer, reaching the underlying glass substrate, the profilometric study reported in Figure S4 was performed. Measurements were acquired using a Dektak 6M Stylus Profiler (Digital Instrument, VEECO). Cross-sections of the channels (red and blue profiles in Figure S4) reveal that material was gradually removed by increasing the etching time up to 90 min. No further increase in depth was observable at longer immersion times. Unfortunately, the absolute values of the crater depth indicated by the profilometer cannot be considered very reliable due to the different stiffness of the irradiated and non-irradiated areas [82-83] which leads to inaccurate measurements, but they qualitatively indicate that the etching process was successful [84].

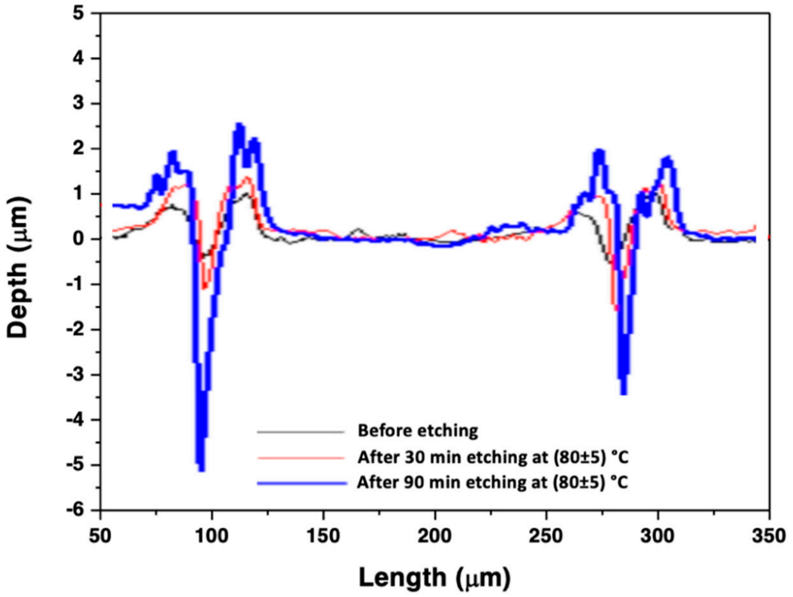

Figure S4. Profilometric analysis of microchannels during wet etching in NaOH at increasing incubation times.

Supplementary Materials section S3: Scanning Electron Microscopy

Scanning Electron Microscopy (SEM) images were acquired on PDMS channels written by PBW and treated with NaOH etchant solution (30% wt) under temperature and time-controlled conditions ((80±5)°C and 90 minutes). Since the PDMS is a non-conductive material, a metallization is required to avoid charging of the sample and so to facilitate the images acquisition during the scansions, without compromising the sample morphology. For this reason, a double layer of chromium and gold of 3 and 5 nm, respectively, was used to cover, by thermal evaporation, the PDMS channels. The chromium is needed to make the next layer of gold adhere better.

Electron microscopy (performed by using SEM, Zeiss Sigma 300 VP) was exploited to image the profiles of the drawn geometry at a closer look that optical imaging. Thus, while optical images in Figures S5 (a) and (b) enable to observe directly the evolution of etching in terms of removal of the proton-beam irradiated PDMS, SEM images in Figures S5(c) and (d) show the regular and stepped profiles allowed by proton beam writing that are clearly observable despite the residuals of metallization applied to remove charging of the insulating sample under electron beam irradiation.

Indeed, a relevant advantage of PBW is that MeV proton beams go through matter along straight paths by keeping their focusing over micro-meter scale distances with low scattering and a deep linear energy transfer. All of this enables to obtain well defined geometries without the grading effects of geometries carried out by wet etching.

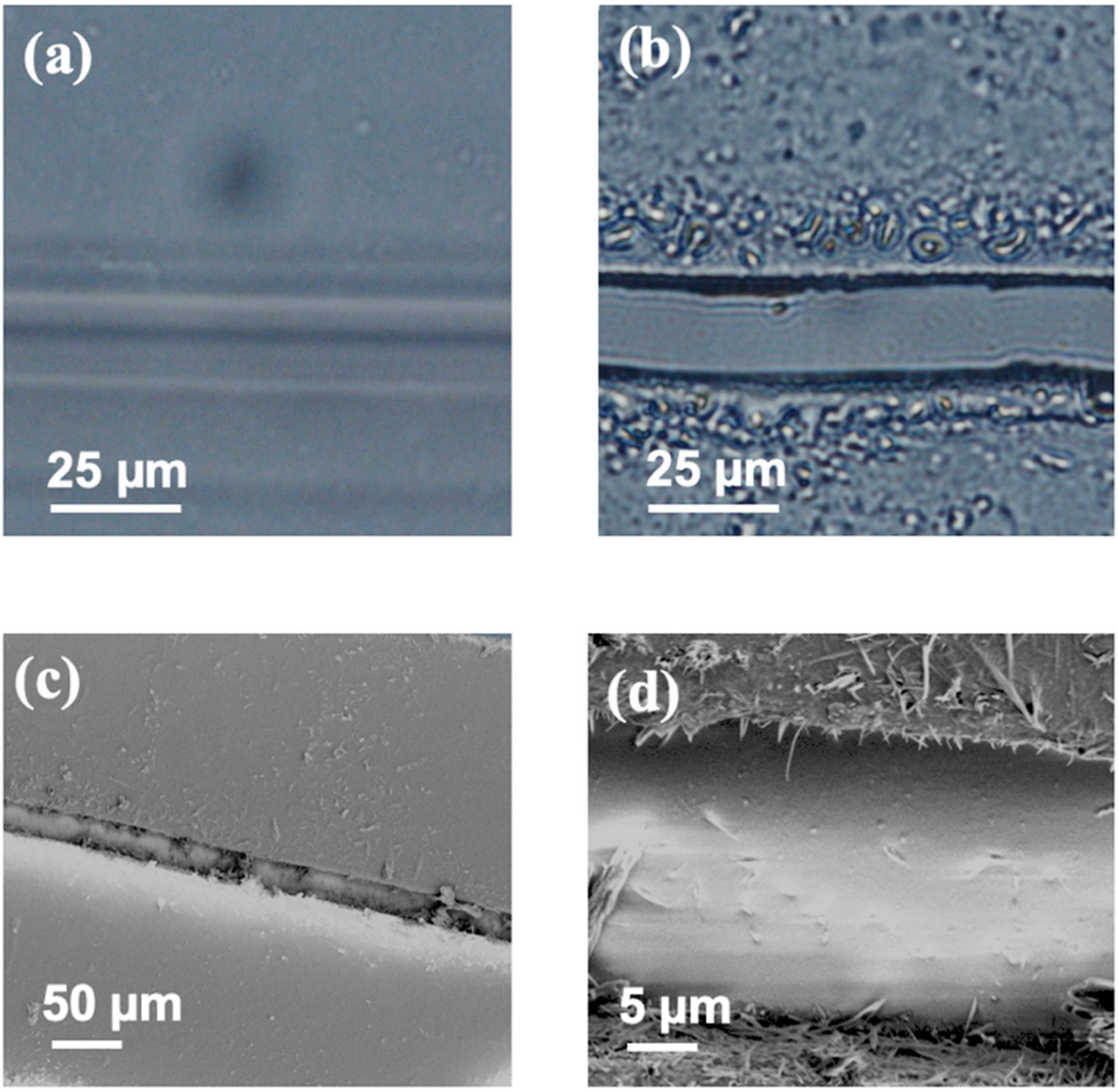

Figure S5. (a)-(b) Optical images of the proton beam written PDMS channels on bulk PDMS (a) before wet etching and (b) after wet etching in a NaOH solution (30% wt) at T = (80±5) °C and incubation times of 90 minutes. (c)-(d) Scanning Electron Microscopy images of the proton beam written PDMS channels on bulk PDMS after wet etching in a NaOH solution (30% wt) at T = (80±5) °C and incubation times of 90 minutes.

Supplementary Materials section S4: Atomic Force Microscopy analysis

Atomic Force Microscopy (AFM) measurements were performed on a 10 µm thick PDMS membrane deposited on top of a glass PEDOT-covered coverslip, which serves as a sacrificial layer during the steps of membrane integration into the final device. The sample was prepared after cleaning a glass substrate with piranha solution (3:1 H_2SO_4:H_2O_2), washing with milli-Q water and drying under nitrogen flow. Then, a thin layer of CLEVIOS PH 500 solution (filtered through the PES syringe filter) was deposited by spin coating followed by baking on a hot plate at 120 °C for 5 min. Finally, a 10 µm thick PDMS membrane was spin-coated by depositing a PDMS solution onto the glass substrate covered by the PEDOT sacrificial layer. The PDMS solution was obtained

from a mixture of pre-polymer/curing agent solution (10:1 weight ratio) diluted with toluene (4:1 in weight). The spin-coated 10 µm thick PDMS layer was cured in an oven at 60 °C overnight.

AFM images were acquired by a Park instrument (XE-100) in non-contact mode, using rectangular silicon probes with a tip radius of 10 nm. For each sample, different areas were scanned to ensure the reproducibility of the results. Image post-acquisition processing was performed by the XEI software. The average roughness (Ra) values were calculated on an area of 10 µm x 10 µm.

AFM measurements demonstrated a Ra of (2.20 ± 0.76) nm for the side of the PDMS membrane over which cells were seeded (Figure S6).

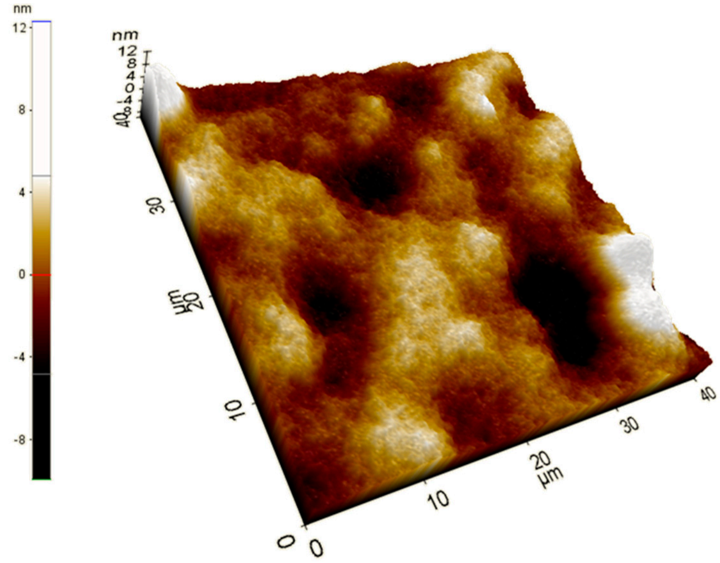

Figure S6. 3D image from an AFM acquisition of the PDMS membrane deposited on top of a PEDOT-coated glass coverslip over an area of 40 µm x40 µm.

Supplementary Materials section S5: Light microscopy imaging of the reference samples

To evaluate if the PBW-based patterning method above-described can be used to fabricate porous membranes serving as substrates for *in vitro* models, the manufactured PBW-holed membrane was integrated into the transwell-like device of interest in this study. It was placed on a slide and 15 thousand Human Cerebral Microvascular Endothelial Cells (HCMEC/D3) were seeded on the opposite side of the membrane and left in culture for 24 hours. The successful issue of the designed drilling and etching protocol was tested by imaging the adhesion of cells on the bottom coverslip. As Figure S7 shows, the PBW-based porous membrane allowed the cells migration between the two compartments of the device.

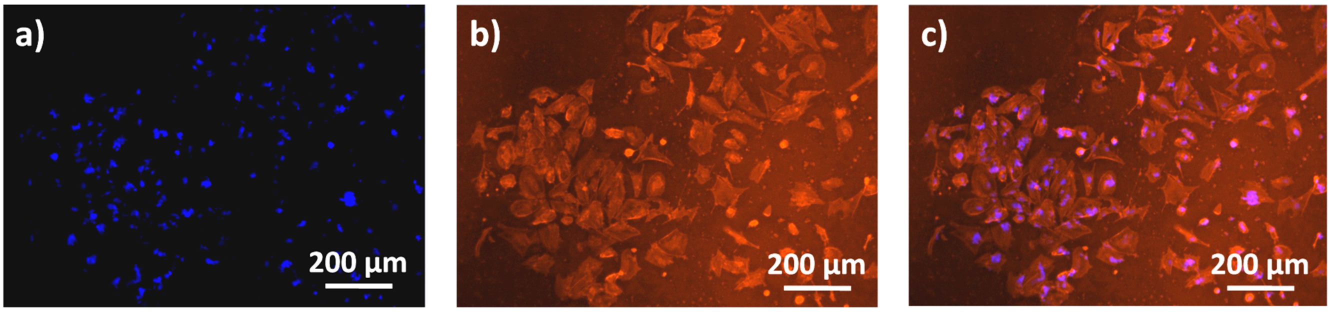

Figure S7. HCMEC/D3 cells on the flat glass surface located at the bottom side of the chip including the PBW-holed PDMS membrane. (a) DAPI channel, (b) phalloidin-TRITC channel, (c) merge of phalloidin-TRITC and DAPI channels.

Supplementary Materials section S6: Image processing and statistical analysis

Figures S8 and S9 show the monogenic signal analysis of the images in Figures 8 (a) and (b) of the main paper. Panels (a) report bright field images merged with phalloidin-TRITC (tetramethylrhodamine isothiocyanate) and DAPI (4',6-diamidino-2-phenylindole) channels showing the distribution of the HCMEC/D3 cells around a PBW-induced single-hole and multiple PBW-processed holes. Panels (b) show the actin filaments following cells stained with phalloidin-TRITC associated with the images of panels (a). Panels (c) and (d) show the spatial maps of the monogenic orientation; panels (e) and (f) show the monogenic phase; panels (g) and (h) show the contrast-enhanced monogenic modulus.

A very first comment suggested by Figures S8 and S9 is that the monogenic phase of the actin filaments is more effective than the bright field images merged with phalloidin-TRITC and DAPI channels to image the cell orientational texture.

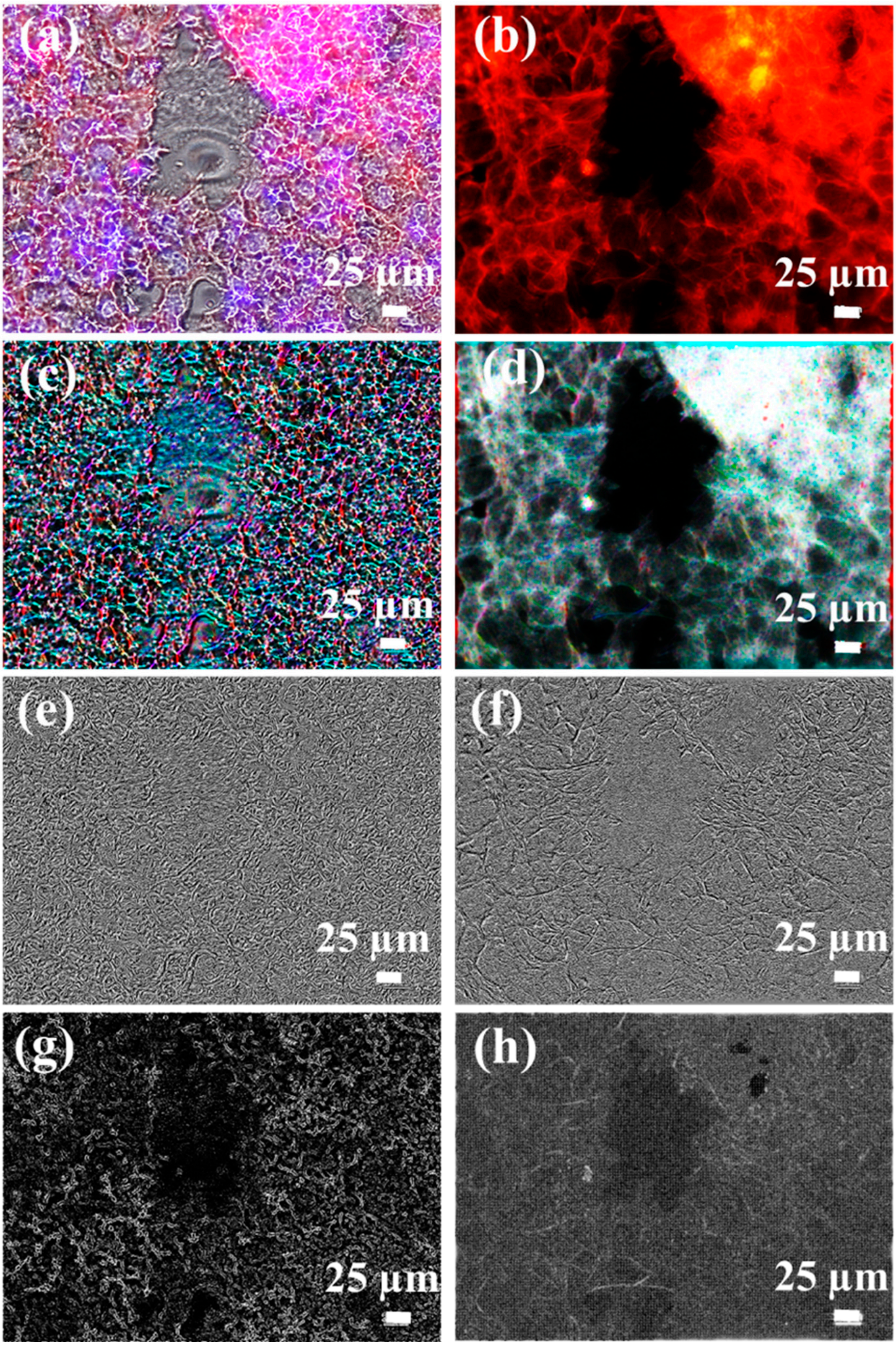

Figure S8. (a) Bright field image merged with phalloidin-TRITC and DAPI channels showing the cell arrangement around a PBW-induced hole. (b) Image of cells stained with phalloidin-TRITC showing the actin filaments distribution around a PBW-induced hole. (c), (e), (g) Spatial maps of the monogenic orientation, phase and modulus associated with the image in panel (a). (d), (f), (h) Spatial maps of the monogenic orientation, phase and modulus associated with the image in panel (b).

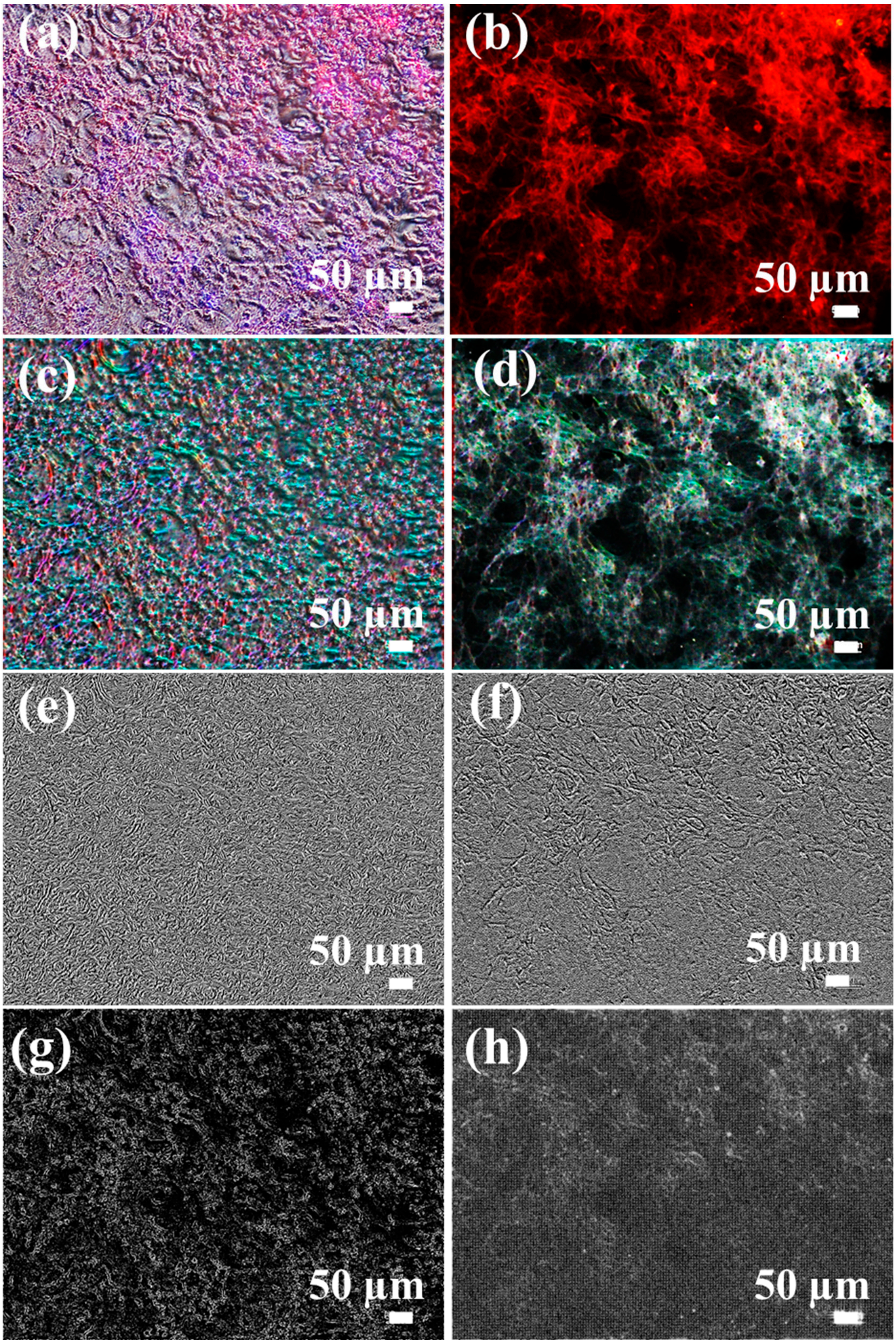

Figure S9. (a) Bright field image merged with phalloidin-TRITC and DAPI channels showing the distribution of the HCMEC/D3 over the PBW-processed holes. (b) Image of cells stained with phalloidin-TRITC showing the actin filaments distribution over the PBW-holed membrane. (c), (e), (g) Spatial maps of the monogenic orientation, phase and modulus associated with the image in panel (a). (d), (f), (h) Spatial maps of the monogenic orientation, phase and modulus associated with the image in panel (b).

In the main paper, it was reported that unperturbed high-density cells have actin filaments strictly aligned with each other locally by digital image processing of a single image for the sake of brevity. As a matter of fact, a systematic analysis, focusing on the distribution of orientations, was performed over multiple regions of interest (ROIs) randomly selected over the surface of a large-area microscopy image of the prepared PBW-holed membrane (Figure S10 (a)). For each ROI, the ellipse method allowed to draw an ellipse or a circle depending on the local anisotropy or isotropy of the image. An ellipse is characterized by different minor and minor axes and a specific orientation that represents the average orientation of the ROI under consideration. Scanning of the source image by the ellipse method demonstrates that locally several ellipses form (red geometrical Figure), which is indicative of cell alignment along the specific direction of the major axis of the ellipse. Anisotropy characteristics are also confirmed by the energy map reported in Figure S10 (c) that shows white high energy features with anisotropic orientational distribution (polar plot in Figure S10 (d)).

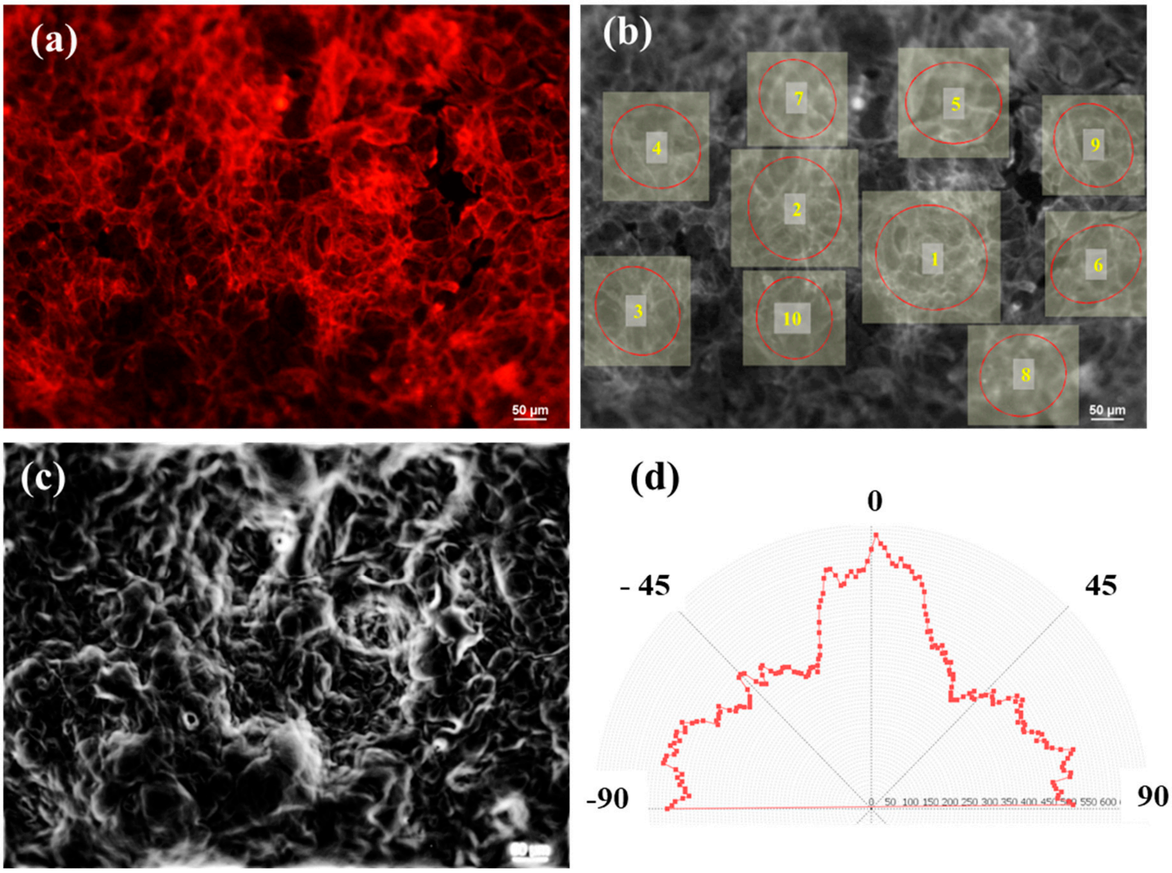

Figure S10. HCMEC/D3 cells on devices integrating a PBW-holed membrane. (a) Light microscopy image of cells stained with phalloidin TRITC showing the actin filaments distribution stained in red color. (b) Local analysis tracking the occurrence of preferential orientations of the cells over randomly distributed ROIs. (c) Energy map associated with the image in panel (a) calculated by the tensor- structure operator. (d) Polar plot of the distribution of orientational anisotropy associated with thewhite features in the energy map shown in panel(c).

List of abbreviations

Atomic Force Microscopy (AFM)

4',6-diamidino-2-phenylindole (DAPI)

Focused Ion Beam (FIB)

Human Cerebral Microvascular Endothelial Cells (HCMEC)

Lab on Chip (LoC)

Organ on Chip (OoC)

Polydimethylsiloxane (PDMS)

Proton Beam Writing (PBW)

Region of interest (ROI)

Scanning Electron Microscopy (SEM)

Stopping and Range of Ions in Matter (SRIM)

Tetramethylrhodamine isothiocyanate (TRITC)

References

73. Van Kan, J.A.; Bettiol, A.A.; Watt, F. Three-dimensional nanolithography using proton beam writing. *Appl. Phys. Lett.* **2003**, *83*, 1629–1631. https://doi.org/10.1063/1.1604468.
74. Furuta, Y.; Nishikawa, H.; Satoh, T.; Ishii, Y.; Kamiya, T.; Nakao, R.; Uchida, S. Applications of microstructures fabricated by proton beam writing to electric-micro filters. *Nucl. Instrum. Methods Phys. Res. Sect. B* **2009**, *267*, 2285–2288. https://doi.org/10.1016/j.nimb.2009.03.037.
75. Udalagama, C.N.B.; Bettiol, A.A.; Watt, F. A Monte Carlo study of the extent of proximity effects in e-beam and p-beam writing of PMMA. *Nucl. Instrum. Methods Phys. Res. Sect. B* **2007**, *260*, 384–389. https://doi.org/10.1016/j.nimb.2007.02.113.
76. Watt, F.; Breese, M.B.H.; Bettiol, A.A.; van Kan, J.A. Proton beam writing. *Mater. Today* **2007**, *10*, 20–29. https://doi.org/10.1016/S1369-7021(07)70129-3.
77. Mistry, P.; Gomez-Morilla, I.; Grime, G.W.; Webb, R.P.; Gwilliam, R.; Cansell, A.; Merchant, M.; Kirkby, K.J.; Teo, E.J.; Breese, M.B.H.; et al. New developments in the applications of proton beam writing. *Nucl. Instrum. Methods Phys. Res. Sect. B* **2005**, *237*, 188–192. https://doi.org/10.1016/j.nimb.2005.04.099.
78. Cutroneo, M.; Havranek, V.; Torrisi, L.; Svecova, B. Ion Micro Beam, promising methods for interdisciplinary research, *J. Inst.* **2016**, *11*, C05001. https://doi.org/10.1088/1748-0221/11/05/C05001.
79. Schmidt, B.; Wetzig, K. *Ion Beams in Materials Processing and Analysis*; Springer: Vienna, Austria, 2013. https://doi.org/10.1007/978-3-211-99356-9.
80. Calcagnile, L.; Quarta, G.; D'Elia, M.; Muscogiuri, D.; Maruccio, L.; Butalag, K.; Gianfrate, G.; Sanapo, C.; Toma, U. Instrumental developments at the IBA-AMS dating facility at the University of Lecce. *Nucl. Instrum. Methods Phys. Res. Sect. B* **2005**, *240*, 22–25. https://doi.org/10.1016/j.nimb.2005.06.081.
81. Podaru, N.C.; Mous, D.J.W. Recent developments and upgrades in ion source technology and ion beam systems at HVE. *Nucl. Instrum. Methods Phys. Res. Sect. B* **2016**, *371*, 137–141. https://doi.org/10.1016/j.nimb.2015.10.021.
82. Satti, A.J.; Andreucetti, N.A.; Ciolino, A.E.; Vitale, C.; Sarmoria, C.; Vallés, E.M. Molecular weight changes induced in an anionic polydimethylsiloxane by gamma irradiation in vacuum. *Radiat. Phys. Chem.* **2010**, *79*, 1137–1143. https://doi.org/10.1016/j.radphyschem.2010.07.003.
83. Huszank, R.; Szilasi, S.Z.; Szikra, D. Ion-Energy Dependency in Proton Irradiation Induced Chemical Processes of Poly(dimethylsiloxane). *J. Phys. Chem. C* **2013**, *117*, 25884–25889. https://doi.org/10.1021/jp406984d.
84. Szilasi, S.Z.; Huszank, R.; Csik, A.; Cserháti, C.; Rajta, I. PDMS patterning by proton beam. *Nuclear Instruments and Methods in Physics Research B* **2009**, *267*, 2296. https://doi.org/10.1016/j.nimb.2009.03.038.
